# Remote Blood Pressure Monitoring With Social Support for Patients With Hypertension: A Randomized Clinical Trial

**DOI:** 10.1001/jamanetworkopen.2024.13515

**Published:** 2024-06-03

**Authors:** Shivan J. Mehta, Kevin G. Volpp, Andrea B. Troxel, Joseph Teel, Catherine R. Reitz, Alison Purcell, Humphrey Shen, Kiernan McNelis, Christopher K. Snider, David A. Asch

**Affiliations:** 1 Department of Medicine, Perelman School of Medicine, University of Pennsylvania, Philadelphia; 2 Center for Health Care Innovation, University of Pennsylvania, Philadelphia; 3 Center for Health Incentives and Behavioral Economics, Leonard Davis Institute of Health Economics, University of Pennsylvania, Philadelphia; 4 Center for Health Equity Research and Promotion, Philadelphia VA Medical Center, Philadelphia; 5 Division of Biostatistics, Department of Population Health, New York University Grossman School of Medicine, New York; 6 Department of Family Medicine and Community Health, Perelman School of Medicine, University of Pennsylvania, Philadelphia

## Abstract

**Importance:**

Hypertension management has traditionally been based on office visits. Integrating remote monitoring into routine clinical practices and leveraging social support might improve blood pressure (BP) control.

**Objective:**

To evaluate the effectiveness of a bidirectional text monitoring program focused on BP control and medication adherence with and without social support in adults with hypertension.

**Design, Setting, and Participants:**

This randomized clinical trial included adults aged 18 to 75 treated at an academic family medicine practice in Philadelphia in 2018 and 2019. Patients had been seen at least twice in the prior 24 months and had at least 2 elevated BP measurements (>150/90 mm Hg or >140/90 mm Hg for patients aged 18-59 years or with diabetes or chronic kidney disease) during visits. All participants had a cell phone with text messaging, offered at least 1 support partner, and were taking maintenance medications to treat hypertension. Patients were randomized 2:2:1 to remote monitoring of BP and medication adherence (RM), remote monitoring of BP and medication adherence with feedback provided to a social support partner (SS), or usual care (UC). Data were analyzed on an intention-to-treat basis between October 14, 2019, and May 30, 2020, and were revisited from May 23 through June 2, 2023.

**Interventions:**

The RM and SS groups received an automatic home BP monitor, 3 weekly texts requesting BP measurements, 1 weekly text inquiring about medication adherence, and a weekly text with feedback. In the SS arm, support partners received a weekly progress report. The UC group received UC through their primary care practice. Clinicians caring for the patients in the intervention groups received nudges via electronic health records to adjust medications when 3 of 10 reported BP measurements were elevated. Patients were followed up for 4 months.

**Main Outcomes and Measures:**

The primary outcome was systolic BP at 4 months measured during the final follow-up visit. Secondary outcomes included achievement of normotension and diastolic BP.

**Results:**

In all, 246 patients (mean [SD] age, 50.9 [11.4] years; 175 females [71.1%]; 223 Black individuals [90.7%] and 13 White individuals [5.3%]) were included in the intention-to-treat analysis: 100 patients in the RM arm, 97 in the SS arm, and 49 in the UC arm. Compared with the UC arm, there was no significant difference in systolic or diastolic BP at the 4-month follow-up visit in the RM arm (systolic BP adjusted mean difference, −5.25 [95% CI, −10.65 to 0.15] mm Hg; diastolic BP adjusted mean difference, −1.94 [95% CI, −5.14 to 1.27] mm Hg) or the SS arm (systolic BP adjusted mean difference, −0.91 [95% CI, −6.37 to 4.55] mm Hg; diastolic BP adjusted mean difference, −0.63 [95% CI, −3.77 to 2.51] mm Hg). Of the 206 patients with a final BP measurement at 4 months, BP was controlled in 49% (41 of 84) of patients in the RM arm, 31% (27 of 87) of patients in the SS arm, and 40% (14 of 35) of patients in the UC arm; these rates did not differ significantly between the intervention arms and the UC group.

**Conclusions and Relevance:**

In this randomized clinical trial, neither remote BP monitoring nor remote BP monitoring with social support improved BP control compared with UC in adults with hypertension. Additional efforts are needed to examine whether interventions directed at helping patients remember to take their BP medications can lead to improved BP control.

**Trial Registration:**

ClinicalTrials.gov Identifier: NCT03416283

## Key Points

**Question:**

Can remote monitoring of blood pressure (BP) alone or with feedback to the patient’s social support partner improve BP control among patients with hypertension?

**Findings:**

In this randomized clinical trial of 246 patients with hypertension, remote BP monitoring and remote BP monitoring with social support did not improve BP control compared with usual care.

**Meaning:**

Findings of this trial indicate that neither remote monitoring of BP alone nor combined with social support result in improved BP control in adults with hypertension. Additional research on interventions aimed at reminding patients to take their BP medications is warranted.

## Teaser

This randomized clinical trial examines whether remote monitoring of blood pressure and medication adherence, with or without feedback to a social support partner, is associated with better control than usual care in adults with hypertension.

## Introduction

Hypertension (HTN) affects about 30% of US adults.^1^ Effective treatment that reduces long-term risk is available, but only about half of adults maintain good blood pressure (BP) control depending on the guidelines used,^2,3,4,5^ and Black patients typically have worse outcomes.^6,7^ Control of HTN requires diagnosis, initiation of treatment, adherence to medications, and titration of medications, traditionally delivered through face-to-face primary care visits.

New care delivery models might improve outcomes. First, substantial literature has shown the benefit of remote monitoring interventions in controlling HTN.^8,9,10,11^ Second, text messaging has become a common form of communication and could be used to engage patients in HTN management.^12,13^ Third, new approaches to clinical practice design that use strategies from behavioral science might make patient engagement more effective.^14,15^ For example, providing motivational feedback to patients might overcome present bias (defined as overvaluing immediate costs or rewards compared with long-term consequences) by highlighting the benefits of taking HTN medications that may not be immediately apparent for this disease, which often has no immediate symptoms.^16^ Additionally, people are influenced by the behavior of others through social accountability (defined as being influenced by connections to other individuals),^17,18^ and an opportunity to engage a friend or family member who might serve as a witness to behavior and to whom the patient might feel accountable may yield better clinical outcomes.^19,20,21^ Evidence of the benefit of a feedback partner is limited, but such benefit could be enhanced through remote monitoring and text messaging in an approach called facilitated cheerleading, in which the technology platforms helps to communicate and enhance social support.^22,23,24^

Among patients with poorly controlled HTN at an academic urban family medicine practice, we evaluated the effect of monitoring BP and medication adherence via bidirectional text messaging with feedback to the patient and, if needed, the clinician. We also compared the effect of remote monitoring combined with providing feedback to a social support partner with providing remote monitoring alone.

## Methods

### Study Design

This 3-arm randomized clinical trial compared the effectiveness of 3 different approaches to improving BP control outside of office visits. Patients were randomly assigned to 1 of 3 study arms: remote monitoring of BP and medication adherence (RM), remote monitoring of BP and medication adherence with feedback to a social support partner (SS), and usual care (UC). The trial was approved by the Institutional Review Board at the University of Pennsylvania. Patients provided informed consent prior to enrollment. The protocol and statistical analysis plan are provided in Supplement 1. This study followed the Consolidated Standards of Reporting Trials (CONSORT) reporting guideline.

### Study Population

We included patients aged 18 to 75 years with a diagnosis of HTN who had visited an urban primary care practice in Philadelphia at the University of Pennsylvania at least twice in the prior 2 years. Patients must have had at least 2 BP readings exceeding the Eighth Joint National Committee (JNC 8) HTN guidelines (>150/90 mm Hg or >140/90 mg/Hg for patients aged 18-59 years or with diabetes or chronic kidney disease) during that time, including at the most recent visit.^25^ Initial BP measurements were obtained from the electronic health record (EHR) during office visits as recorded by routine clinical practice. To be included in the trial, patients needed to have a cell phone with text messaging capability, offer at least 1 support partner, and be taking at least 1 of the JNC 8–recommended medications for HTN. We excluded patients if they had evidence of metastatic cancer, end-stage kidney disease, congestive heart failure, dementia, or a body mass index (calculated as weight in kilograms divided by height in meters squared) of 50 or greater.

### Recruitment and Randomization

Eligibility was confirmed by study staff via manual review of automated data extracted from the EHR. All eligible patients were mailed recruitment letters and an informational brochure followed by up to 5 phone calls from study staff. Included patients completed a short survey assessing their current BP monitoring and medication adherence rates and forgetfulness about taking medication.

Patients were randomly assigned to the RM, SS, or UC arm in a 2:2:1 ratio using variably sized permuted blocks of 5 and 10. Randomization was conducted using the Way to Health platform, a software platform that facilitates and automates many aspects of study design and intervention implementation.^26^ Primary care clinicians were notified by a note in the EHR when one of their patients was enrolled in the study. For patients randomly assigned to the SS arm, support partners identified during recruitment were contacted and their participation was requested in support of the patient. Enrolled support partners provided their assent for participation.

The study was conducted in 2 phases. Patients in phase 1 were enrolled between May 4 and August 3, 2018; the end-of-study visit was on December 15, 2018. A total of 151 patients were recruited in phase 1. Patients in phase 2 were enrolled between January 2 and March 27, 2019; the end-of-study visit was on August 8, 2019. A total of 100 patients were recruited in phase 2. Patients received $25 upon enrollment and an additional $50 for completing the follow-up appointment at the end of the 4-month study period. Investigators and data analysts were blinded to arm assignment, but patients and research staff were not.

### Interventions

Patients in both the RM and SS arms were mailed an electronic BP cuff (model BP710N; OMRON Healthcare, Inc). An adult size, extra-large cuff was provided upon patient request (Medline). Patients received 3 text prompts per week to take and submit their BP measurements, and 1 text each week asking them about their medication adherence for the past week. Patients in both of these arms also received a weekly text with feedback on their BP monitoring and medication adherence from the study staff. Additionally, in the SS arm, support partners received a weekly text update on the BP monitoring and medication adherence of their associated patient. Support partners were able to opt out of providing feedback to their associated patient. If the support partner did not opt out, the patient received a once-weekly feedback text message on behalf of their support partner either encouraging them to continue their good work or to try to do better in the following week, depending on their performance (trial protocol in Supplement 1). Patients in the RM and SS arms were monitored for 4 months. Patients randomly assigned to the UC control arm received UC as provided by the clinical practice, which included office visits only as scheduled through routine practice.

For phase 1 (151 patients), if at any point 3 of 10 reported BP measurements were elevated per JNC 8 guidelines, the elevated measurements and any subsequent measurements (up to 10) were sent via EHR message to the patient’s primary care physician (PCP) along with the patient’s reported medication adherence and a nudge to adjust medications (“JNC 8 guidelines suggest that hypertension medications should be adjusted and added until blood pressure is controlled.”). Measurements were reported again in this same manner if they remained elevated and at least 3 weeks elapsed between nudges.

If at any time a patient’s BP was severely elevated (systolic BP ≥180 mm Hg or diastolic BP ≥110 mm Hg), a text message was sent to the patient with instructions to recheck in 15 minutes and if the BP remained elevated, to call the clinic to discuss their measurement with a nurse or other clinician. This severely elevated BP measurement, along with information about patient-reported medication adherence, was also reported directly to the PCP via an EHR message.

Based on feedback from clinicians, this procedure was updated for phase 2 (100 patients). At this time, the study team implemented an integration between the Way to Health platform and patient EHRs, so that all patient-reported BP measurements populated into a flowsheet in the EHR, and a centrally designated team of nurses and a nurse practitioner (NP) were identified to manage patient follow-up and medication changes. On enrollment into 1 of the 2 intervention arms, in addition to notifying the PCP of the patient’s enrollment, research staff sent a special BP monitoring order to the NP, who approved and signed the order for each patient. This order electronically authorized patient-submitted BP measurements to be reviewed via flowsheet directly within the patient EHR. Nursing staff met twice weekly with the NP to review all monitoring notifications in bulk and make appropriate medication adjustments with follow-up phone calls or visits as needed. Any changes to patient medication were then routed via the EHR to the PCP.

### End-of-Study Visits

All patients were invited to an in-person follow-up visit 4 months after enrollment, scheduled between 30 days before and 30 days after the target date. Study staff performed the follow-up BP measurement at the practice site using the same machines used for routine office visits (Welch Allyn model 4200B). Patients rested for 5 minutes prior to having their BP measured, during which time they completed a brief follow-up survey assessing end of study BP monitoring and medication adherence and their experience with the intervention as measured by a net promoter score. The net promoter score measures the likelihood of a patient to recommend a service on a score of 1 (not likely) to 10 (very likely). Values of 7 and 8 are discarded and the number of detractors (6 and below) is subtracted from the number of promoters (9 or 10) to calculate the score (range, –100 to 100). Blood pressure was measured 3 times with a 1-minute rest between each measurement. The second and third BP measurements were averaged and recorded as the final BP measurement of the study. This measurement was routed as an encounter to the nursing pool and to the PCP via the EHR as a final study closeout.

### Study Outcomes

The primary outcome was the systolic BP at the 4-month visit according to the intervention arm. Secondary outcomes included achievement of normotension (blood pressure control) and diastolic BP. We also evaluated self-reported BP and medication adherence submissions during the intervention. Race and ethnicity was based on self-reported data in the EHR as Black, Hispanic, White, other (patients who self-identified as other race), or unknown.

### Statistical Analysis

Using the intention-to-treat approach, the primary analysis evaluated the systolic BP at the 4-month visit to the trial arm, adjusting for the initial systolic BP by including the baseline measures in the model. For patients with missing BP data, we first included BP measurements available from the EHR occurring from 90 to 150 days after the participant’s enrollment in the trial, and then conducted multiple imputation using all available baseline covariates (all 246 participants). Secondary analyses assessed achievement of BP control by trial arm using χ^2^ tests and repeated the analyses for systolic BP with diastolic BP at the 4-month visit (all 246 participants). In addition, we tracked BP measurements by arm from the EHR that were obtained through UC for up to 8 months after the end of the trial. We also compared self-reported BP monitoring and medication adherence by trial arm at baseline and at the end-of-study visit (209 patients) based on patient survey responses.

Systolic and diastolic BPs were compared using multivariable linear regression, and BP control was evaluated using χ^2^ tests, with a 2-sided *P* < .02 considered significant. The mean percentage of expected BP measurements received for patients in the RM and SS arms was compared against the UC arm using an independent *t* test with a 2-sided *P* < .05 considered significant. All statistical analyses were performed using R, version 4.0.3 (R Project for Statistical Computing), with multiple imputation performed using the mice package in R.^27^

Assuming a systolic BP SD of 5.3 mm Hg (given variability of BP over time) and a 2-sided significance level of *P* < .02 (to accommodate the 3 pairwise comparisons), the sample size of 60 patients in each intervention arm and 30 patients in the control arm provided 80% power to detect a difference in systolic BP of 3.75 mm Hg between either the RM or SS group and the UC group, and a difference in systolic BP of 3.10 mm Hg between the RM and SS arms. However, based on additional clinical information obtained after the study was initiated, we estimated an SD for systolic BP of 20 mm Hg, larger than our initial estimate. Thus, we increased our accrual target to 100 patients in each intervention arm and 50 patients in the control arm, which provides 80% power to detect a difference in systolic BP of 11 mm Hg between either the RM or SS groups and the UC group, and a difference in systolic BP of 9 mm Hg between the RM and SS arms. All analyses were conducted between October 14, 2019, and May 30, 2020, and were revisited from May 23 through June 2, 2023.

## Results

### Patient Characteristics

We contacted 810 eligible patients identified through automated data extraction from the EHR from April 2018 to October 2018. In all, 251 patients enrolled in the trial and were randomly assigned, with 101 patients assigned to the RM arm, 100 to the SS arm, and 50 to the UC arm (Figure). A total of 246 patients (mean [SD] age, 50.9 [11.4] years; 175 females [71.1%] and 71 males [28.9%]; 223 Black patients [90.7%], 1 Hispanic or Latino patient [0.4%], 13 White patients [5.3%], 6 patients [2.4%] of other races, and 5 patients [2.0%] of unknown race and ethnicity) were included in the intention-to-treat analysis: 5 of the enrolled patients were excluded (2 patients were ineligible, 1 left the practice, 1 withdrew from the trial, and 1 died of unrelated causes). A total of 151 patients were enrolled in phase 1 and 100 in phase 2: 100 patients in the RM arm, 97 in the SS arm, and 49 in the UC arm. While 213 of 246 patients (86.6%) attended the end-of-study visit, 3 were excluded from complete case analyses because their last visit was outside of the 30-day window, and 4 more were excluded because of incomplete BP data from the visit. Of the 206 patients (83.7%) with complete end of study data, we found follow-up visit completion was higher in the 2 intervention arms (84.0% in the RM arm and 89.6% in the SS arm) than in the UC arm (71.4%). Additionally, 85 patients (34.6%) had diabetes and 26 (10.6%) had chronic kidney disease (Table 1). We were able to enroll 88 of 100 support partners in the SS arm.

**Figure.**
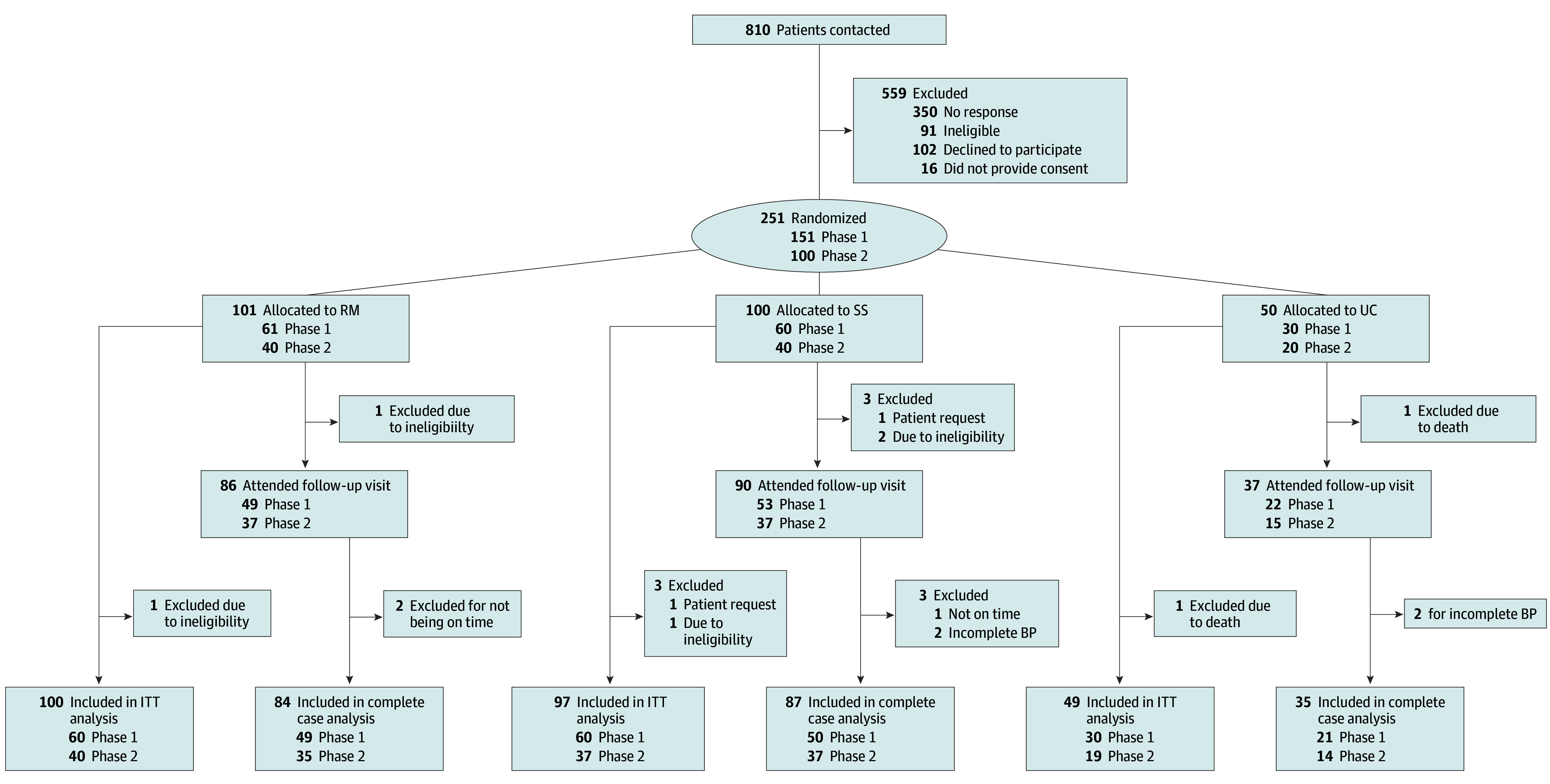
Trial Flow Diagram BP indicates blood pressure; ITT, intention to treat; RM, remote monitoring of BP and medication adherence; SS, remote monitoring of BP and medication adherence with feedback to a social support partner; and UC, usual care.

**Table 1.** Participant Characteristics in Intention-to-Treat Analyses

Characteristic	Participants, No. (%)		
	RM arm (n = 100)	SS arm (n = 97)	UC arm (n =49)
Age, mean (SD), y	50.7 (10.1)	52.2 (12.5)	49.1 (11.5)
Sex			
Female	74 (74.0)	65 (67.0)	36 (73.5)
Male	26 (26.0)	32 (33.0)	13 (26.5)
Race			
Black	92 (92.0)	83 (85.6)	48 (98.0)
White	3 (3.0)	9 (9.3)	1 (2.0)
Other^a^	5 (5.0)	1 (1.0)	0
Unknown	0	4 (4.1)	0
Ethnicity			
Hispanic or Latino	1 (1.0)	0	0
Not Hispanic or Latino	99 (99.0)	96 (99.0)	49 (100)
Unknown	0	1 (1.0)	0
Health insurance			
Medicaid	27 (27.0)	22 (22.7)	19 (38.8)
Medicare	15 (15.0)	32 (33.0)	5 (10.2)
Private	56 (56.0)	41 (42.3)	25 (51.0)
None	2 (2.0)	1 (1.0)	0
Unknown	0	1 (1.0)	0
Comorbidities			
Chronic kidney disease	9 (9.0)	13 (13.4)	4 (8.2)
Diabetes	35 (35.0)	37 (38.1)	13 (26.5)
First baseline systolic BP, mean (SD), mm Hg	152.5 (12.7)	149.9 (12.7)	151.4 (12.4)
First baseline diastolic BP, mean (SD), mm Hg	90.1 (10.6)	86.5 (10.1)	90.5 (8.6)
Second baseline systolic BP, mean (SD), mm Hg	151.3 (12.9)	150.0 (10.5)	151.1 (11.2)
Second baseline diastolic BP, mean (SD), mm Hg	87.4 (10.5)	88.0 (11.8)	88.9 (8.8)

Abbreviations: BP, blood pressure; RM, remote monitoring; SS; remote monitoring with social support; UC, usual care.

^a^ Includes patients who self-identified as other race.

### Blood Pressure Outcomes

In the primary analysis, which adjusted for baseline systolic BP, systolic BP was not significantly lower in either the RM arm (adjusted mean difference, −5.25 [95% CI, −10.65 to 0.15] mm Hg; *P* = .06) or the SS arm (adjusted mean difference, −0.91 [95% CI, −6.37 to 4.55] mm Hg; *P* = .74) compared with the UC arm at the end of the study (Table 2). The results were similar after adjusting for age, sex, race and ethnicity, body mass index, and diabetes status (Table 3), when we included only participants with complete end of study data (eTable 1 in Supplement 2), or when we examined diastolic BP for the different analyses (Tables 2 and 3; eTable 1 in Supplement 2). Compared with the UC group, there was no significant difference in diastolic BP at the 4-month follow-up visit in the RM arm (diastolic BP adjusted mean difference, −1.94 [95% CI, −5.14 to 1.27] mm Hg) or the SS arm (diastolic BP adjusted mean difference, −0.63 [95% CI, −3.77 to 2.51] mm Hg).

**Table 2.** End of Study BP Adjusted for Baseline BP

	Adjusted mean difference (95% CI)	*P* value^a^
Systolic BP, mm Hg		
Intercept	85.74 (58.66 to 112.83)	
RM arm	−5.25 (−10.65 to 0.15)	.06
SS arm	−0.91 (−6.37 to 4.55)	.74
UC arm	0 [Reference]	NA
Baseline BP	0.33 (0.16 to 0.51)	<.001
Diastolic BP, mm Hg		
Intercept	46.69 (35.42 to 57.96)	
RM arm	−1.94 (−5.14 to 1.27)	.23
SS arm	−0.63 (−3.77 to 2.51)	.70
UC arm	0 [Reference]	NA
Baseline BP	0.43 (0.31 to 0.56)	<.001

Abbreviations: BP, blood pressure; NA, not applicable; RM, remote monitoring; SS; remote monitoring with social support; UC, usual care.

^a^ Threshold for statistical significance is *P* < .02.

**Table 3.** End of Study BP Adjusted for Sociodemographic and Clinical Variables^a^

Variable	Adjusted mean difference (95% CI)	*P* value^b^
**Systolic BP, mm Hg**		
Intercept	77.17 (47.20 to 107.14)	
RM arm	−5.56 (−11.07 to −0.06)	.05
SS arm	−1.83 (−7.50 to 3.84)	.52
UC arm	0 [Reference]	NA
Baseline systolic BP	0.34 (0.14 to 0.54)	.001
Age	0.08 (−0.11 to 0.26)	.41
Sex		
Female	0 [Reference]	NA
Male	4.30 (−0.44 to 9.04)	.08
Race		
Black	0 [Reference]	NA
White	1.06 (−7.35 to 9.47)	.80
Other or unknown	−1.44 (−11.07 to 8.20)	.77
BMI	0.04 (−0.23 to 0.30)	.79
Diabetes		
No	0 [Reference]	NA
Yes	4.30 (−0.05 to 8.65)	.05
**Diastolic BP, mm Hg**		
Intercept	69.31 (49.68 to 88.94)	
RM arm	−2.00 (−5.30 to 1.26)	.77
SS arm	−0.64 (−3.96 to 2.68)	.70
UC arm	0 [Reference]	NA
Baseline diastolic BP	0.30 (0.16 to 0.46)	<.001
Age	−0.17 (−0.28 to −0.05)	.005
Sex		
Female	0 [Reference]	NA
Male	0.78 (−1.54 to 3.09)	.51
Race		
Black	0 [Reference]	NA
White	−0.76 (−5.55 to 4.02)	.75
Other or unknown	−0.13 (−6.43 to 6.17)	.97
BMI	−0.08 (−0.03 to 0.10)	.38
Diabetes		
No	0 [Reference]	NA
Yes	0.31 (−2.09 to 2.71)	.80

Abbreviations: BMI, body mass index (calculated as weight in kilograms divided by height in meters squared); BP, blood pressure; NA, not applicable; RM, remote monitoring; SS; remote monitoring with social support; UC, usual care.

^a^ Adjusted for baseline systolic and diastolic BP, age, sex, race, ethnicity, BMI, and diabetes status.

^b^ Threshold for statistical significance is *P* < .02.

Overall, 48.8% (41 of 84) of patients in the RM arm achieved BP control at the end of the study compared with 31.0% (27 of 87) of patients in the SS arm and 40.0% (14 of 35) of patients in the UC arm, with no statistical difference across arms (Table 4). The eFigure in Supplement 2 reveals favorable shifts in systolic and diastolic BP measurements from the start to the end-of-study visit that are indistinguishable from those in in the UC group. In a post hoc analysis, we did not find any differences in change in systolic BP and proportion of patients with normotension between either intervention and control arms 12 months from the start of the trial based on any office BP measurements in the EHR between 4 and 12 months after the date of enrollment (eTable 4 in Supplement 2). The percentage of expected BP measurements reported was similar between the RM and SS arms (mean [SD], 76.5% [19.8%] and 77.2% [21.8%]; *P* = .82) (eTable 2 in Supplement 2).

**Table 4.** Change in BP and BP Control at End of Study and Between 4 and 12 Months After Enrollment (Complete Cases Only, Unadjusted)

	Trial arm			*P* value^a^		
	RM	SS	UC	RM vs SS	RM vs UC	SS vs UC
**End-of-study visit**						
No. of patients	84	87	35	NA	NA	NA
Change in BP, mean (SD), mm Hg						
Systolic	−19.36 (15.99)	−14.72 (15.56)	−14.91 (14.74)	.06	.15	.95
Diastolic	−4.4 (9.34)	−3.17 (9.09)	−4.3 (9.30)	.39	.96	.54
BP controlled, No. (%)	41 (48.81)	27 (31.03)	14 (40.00)	.03	.50	.46
**At 4-12 mo after enrollment**						
No. of patients^b^	67	71	27	NA	NA	NA
Change in systolic BP, mean (SD), mm Hg	4.69 (17.34)	3.56 (19.30)	−2.59 (17.41)	.71	.07	.13
BP controlled, No. (%)	26 (38.81)	25 (35.21)	11 (40.74)	NA	NA	NA

Abbreviations: BP, blood pressure; NA, not applicable; RM, remote monitoring; SS; remote monitoring with social support; UC, usual care.

^a^ *P* < .02 is considered statistically significant due to 3 pairwise comparisons.

^b^ Number of patients at 4 to 12 months after enrollment is lower than at end of study since not all patients had an ambulatory BP measurement within that window.

There were similar rates of primary care, emergency department, and hospital visits across arms (eTable 3 in Supplement 2). In a post hoc analysis, there was no difference in the primary outcome between phases 1 and 2 (eTable 4 in Supplement 2). The mean (SD) time from enrollment through the end-of-study visit (phases 1 and 2 combined) was 125.2 (7.4) days in the RM arm, 121.0 (6.9) days in the SS arm, and 129.9 (7.6) days in the control arm (eTable 5 in Supplement 2). At enrollment, combination medications (ie, pills containing 2 different medications) were being used by 20 of 101 patients (19.8%) in the RM arm, 20 of 100 patients (20.0%) in the SS arm, and 12 of 50 patients (24.0%) in the UC arm. Patients across arms were taking a similar number of medications at the start of the study (mean [SD], 1.6 [0.7] drugs in the RM arm, 1.7 [0.9] drugs in the SS arm, and 1.5 [0.7] drugs in the UC arm).

Across both phases, requesting an office visit with the patient for BP follow-up was the second most common action taken in response to alerts (21.7%), while the most common response to alerts was to take no action (37.9%). Medications were titrated only 17.4% of the time, in a mix between remote management and in-person visits. When we reviewed clinician actions by study arm, we found that while medication doses in both groups were titrated at similar rates, alerts from the SS arm were acted on less frequently than alerts from the RM arm (38.8% vs 30.9%).

### Follow-up Surveys

Patients self-reported their BP monitoring frequency at baseline and at the end of the study. At baseline, all groups self-reported a median monitoring frequency of 0 during the last 14 days (IQR, 0-2 in the intervention groups and 0-0 in the control group). At the end of the trial, median (IQR) reporting frequency was 9 (4-14) days in the RM arm, 8 (6-14) days in the SS arm, and 0 (0-2) days in the UC arm. All groups self-reported being adherent with medications 14 of 14 days at both baseline and the end-of-study visit (eTable 6 in Supplement 2). However, fewer participants from the intervention arms compared with the UC arm reported difficulty remembering to take their BP medications at the end-of-study visit compared with baseline (eTable 6 in Supplement 2): 27.4% in the RM arm and 19.3% in the SS arm and 43.2% in the UC arm.

Participants in the intervention arms generally agreed that the program helped them to remember to both monitor their BP and to take their medications. Participants in the RM arm were overwhelmingly very likely to recommend the program to a friend or family member who may need it (90.5%). Overall, participants gave the program a net promotor score of 76 of 100 (eTable 7 in Supplement 2).

## Discussion

In this randomized clinical trial, we found no significant improvement in BP control in either of the remote monitoring arms compared with UC. Likewise, there was no difference in self-reported frequency of BP monitoring in the intervention groups with or without social support.

The intervention incorporated remote engagement with text messaging, the provision of home BP monitors, integration of data in the EHR, and social support to help patients improve BP control. Despite the conceptual appeal of these interventions, we found no improvement in BP control in this study. Several factors might explain the results. First, the intervention was primarily focused on patient behavior, while clinician management and prescribing may also influence BP control.^28^ In phase 1, the BP alerts may have overburdened PCPs, who may not have had enough time to respond appropriately. Only in phase 2 were alerts sent to a dedicated team of nurses and NPs. Additionally, there is evidence that clinical inertia may impede dose escalation.^29^

Second, there may not have been enough time in the 4-month intervention for BP control to change. Many of the PCPs were still relying on office visits for responding to BP alerts since the current payment model for primary care at this institution still relies on reimbursement for office visits, which may take weeks or months to schedule. Across both phases, requesting an office visit with the patient for BP follow-up was the second most common action taken in response to alerts (21.7%), while the most common response to alerts was to take no action (37.9%). Medications were titrated only 17.4% of the time, in a mix between remote management and in-person visits. When we reviewed clinician actions by study arm, we found that while medication doses in both groups were titrated at similar rates, alerts from the SS arm were acted on less frequently than alerts from the RM arm (38.8% vs 30.9%).^30^ Nevertheless, we did not observe differences across arms when we extended our assessment of outcomes an additional 8 months after the end of study office visit.

Third, social support partners may have been able to provide social accountability but may not have known how to provide substantive support. Fourth, the study relied on an opt-in consent process, so we may have selected patients who were particularly motivated and may have improved BP with no intervention. Also, the enrollment process and final BP check for the control group may have acted as an intervention. Our control group experienced a 40% rate of controlled BP at the end of the study.

### Strengths and Limitations

The trial has strengths in design and evaluation. This trial was conducted in close partnership with an urban primary care practice with a large proportion of Black patients, who are known to have worse outcomes in BP management. We also leveraged new technology through text messaging and automation that are being used more in practices across the country, particularly since the COVID-19 pandemic. Finally, we rigorously evaluated the utility of creating a mechanism for increased social accountability that holds promise for health care delivery but also needs to be evaluated in additional clinical care settings.

An important limitation of the study is that there may not have been sufficient power to detect smaller improvements in BP control, so we cannot make conclusions about the effectiveness or lack of effectiveness of remote monitoring. The SD of the mean systolic BP change was greater than the initial estimate but lower than what was estimated in the revised power calculation. Additionally, as this was a pragmatic design, the inclusion criteria for BP control were based on routine office visits, while the outcome ascertainment was conducted through a separate end of study research visit using the same equipment, and research staff were unblinded. Finally, there was differential follow-up for the final visit, but we conducted imputation to account for missing data.

## Conclusions

In this randomized clinical trial of adults with hypertension, we found that remote BP monitoring did not result in a statistically significant improvement in BP control with or without social support compared with UC. Future efforts to examine whether interventions directed at helping patients remember to take their BP medications, including additional insights from behavioral science, clinical pathways for dose escalation, and workflow redesign for dedicated staff, could aid in BP control.

## Notes

Supplement 1.
Trial Protocol

Supplement 2. **eTable 1.** End of Study BP (Complete Cases Only, n = 206) **eTable 2.** Adherence to BP Monitoring (Complete Cases Only) **eTable 3.** Mean Visit Utilization During and After BP Monitoring **eTable 4.** Change in Systolic BP Between Baseline and End of Study Adjusted for Baseline Systolic BP Among Phase 1 (2018) and Phase 2 (2019) Participants **eTable 5.** Days Elapsed from Enrollment Through End of Study Visit **eTable 6.** Self-Reported Frequency of BP Monitoring and Medication Adherence at Baseline and End of Study (Paired Cases Only, n = 209) **eTable 7.** Participant Experience (Completed Patients in RM or SS Group) **eFigure.** Changes in BP Distribution from Baseline to End of Study (Complete Cases Only)

Supplement 3.
Data Sharing Statement
