# Supplementary material for: Remote Blood Pressure Monitoring With Social Support for Patients With Hypertension: A Randomized Clinical Trial
Source: JAMA Netw Open. 2024 Jun 3;7(6):e2413515. doi: 10.1001/jamanetworkopen.2024.13515 (PMC11148689; doi:10.1001/jamanetworkopen.2024.13515)
Supplement: Supplement 1. — Trial Protocol [file jamanetwopen-e2413515-s001.pdf]

Initial Protocol

Abstract
A 3-arm randomized pilot trial aimed at comparing the effectiveness of different approaches to improving blood pressure (BP) control: A) remote monitoring of BP and medication adherence; B) remote monitoring of BP and medication adherence with feedback to social support; and C) usual care. The targeted population is patients aged 18-75 within the Penn Family Care practice with at least 2 visits within the prior 12 months with blood pressure readings exceeding recommended HTN guidelines.

Study Instruments
All subjects will complete a baseline and post-intervention survey. The baseline survey will collect basic demographics and baseline medication adherence and blood pressure monitoring frequency. The post-intervention survey will collection similar adherence and monitoring information, as well as qualitative data regarding patient perceptions about the interventions.

Group Modifications
For subjects in the Control (usual care) arm of the study, the subjects will not receive text reminders to monitor their blood pressure or take their medication(s). For subjects in the Remote Monitoring plus Social Support (RM+SS) arm of the study, the subjects will receive additional feedback from a designated support partner.

Administration of Surveys
All subjects will complete a baseline and post-intervention survey. The baseline survey will collect basic demographics and baseline medication adherence and blood pressure monitoring frequency and be conducted over the phone after the participant has been consented. The post-intervention survey will collect similar adherence and monitoring information, as well as qualitative data regarding patient perceptions about the interventions. The post-intervention survey will be completed at the in-person 4 month visit. These surveys should take no more than 15 minutes to complete. Demographic and socio-economic questions are modified from demographic questions on the Behavioral Risk Factor Surveillance System survey, administered by the Centers for Disease Control and Prevention.

Objectives
1.1 Objectives
We plan to leverage the Way to Health (WTH) platform to develop and evaluate a new HTN service-delivery model that is integrated with routine clinical care at Penn Family Care (PFC), an academic family medicine practice in West Philadelphia. Among patients with poorly controlled HTN:

1. We will assess the effects of monitoring blood pressure and medication adherence with feedback to the patient and to the clinical practice, as needed if out of control.
2. We will also compare the impact of providing feedback to a social support partner with what we call facilitated cheerleading.

1.2 Primary Outcome Variable
The primary outcome is systolic blood pressure during the 4-month study visit.

1.3 Secondary Outcome Variable(s)
Secondary outcomes include the percent of patients that are normotensive, estimation of recruitment and attrition rates, and evaluation of patient perceptions about the interventions. We will also compare medication adherence by intervention arm after enrollment, adjusted for BMI, age, gender, race, forgetfulness (from the baseline and follow-up surveys), and diabetic status. In addition, we will track BP

measurements from the EMR that are obtained through usual care as well as during PCP visits, MPM messaging, and phone calls; hospitalizations; and medication changes for up to 9 months after the study intervention using similar models. We will analyze diastolic blood pressure at the 4-month visit, adjusting for the initial diastolic blood pressure.

Background

1.1 Program Goals

Hypertension (HTN) affects about 30% of US adults, and effective treatment that reduces long-term risk is available, but only about half of adults maintain good control. HTN control requires diagnosis, initiation of treatment, adherence to medications, and titration of medications. The traditional visit-based primary care model is inadequate to address management needs, given the limited visit time and lack of insight into the behavior of patients outside of the clinic setting.

Substantial literature has shown the benefit of remote monitoring interventions in controlling HTN, but the effects are not typically sustained and traditional primary care practices may not have the infrastructure to respond to remote monitoring data. There is an opportunity to develop automated approaches to monitoring blood pressure and medication adherence by leveraging principles of behavioral economics and clinical practice re-design. Our focus is on self-monitoring of blood pressure and medication adherence using bi-directional text messaging.

In addition to providing feedback to patients, we will also engage a friend or family member identified by the patient who has agreed to act as a feedback partner. Those with active social support typically have better clinical outcomes, and the feedback partner could serve as a witness to non-adherence as well as a cheerleader. Despite considerable enthusiasm for the concept that social support can be used to advance medication adherence, there is currently no evidence that this provides benefit beyond individual feedback. The tepid results from those few studies may be explained by limited participation of the feedback partner or an insufficient dose of cheerleading. There may be greater impact if we make it easier for them to communicate with the patient to provide motivation. Some health systems have employed a proactive approach to identifying eligible patients and encouraging participation in CRC screening through direct outreach, thereby eliminating barriers to screening such as the in-office visit. Kaiser Permanente Northern California is one such example that improved their screening rates to over 80% through mailed FIT outreach to eligible patients. The opportunity exists to extend this model to other health systems, such as UPHS, to engage in organized direct outreach with mailed FIT in order to enhance CRC screening rates.

In this study, we will be using population-based outreach screening to better understand mailed CRC screening uptake. By evaluating the effectiveness of opt out versus opt in mailed outreach screening, we will enhance the public health capacity, and efficiency, to increase CRC screening uptake and reduce preventable death from this disease. Default settings (such as opt out) have been effective in producing desired behavior actions in a variety of economic and clinical contexts, including employee enrollment in retirement savings, organ donation, subject enrollment in research studies and breast screening. However, there is a lack of evidence for use in CRC screening. Moreover, by furthering our understanding of patient-level facilitators and barriers to completing mailed FIT, we hope to support the need for further research to improve the delivery of CRC screening and screening uptake in diverse population groups.

Statistical Considerations

1.1 Power and sample size

Assuming 5.3mmHg standard deviation of SBP (given variability of BP over time) and a two-sided significance level of 0.017 (to accommodate the three pairwise comparisons), our sample size of 60 in each intervention arm and 30 in the control arm provides 80% power to detect a difference in SBP reduction of 3.75 mmHg between either intervention and usual care, and a difference in SBP reduction of 3.1 mmHg between the remote monitoring and social influence arms. A preliminary data extraction shows about 2300 patients uncontrolled hypertensive patients in PFC, which should provide a sufficient population for enrollment.

1.2 Data analysis

As a randomized controlled trial, we will conduct intention to treat analysis. For patients with missing data, we will first include blood pressure readings from the EMR and then conduct multiple imputations.

The primary outcome will relate the systolic blood pressure during the 4 month visit to intervention arm, adjusting for the initial systolic blood pressure by including baseline measures in the model. For patients with missing data who do not complete the 4 month in-person blood pressure reading, we will conduct multiple imputation of the SBP values at 4 months as a function of baseline variables, including intervention arm.

Secondary analyses include modeling of normotensive status using logistic regression; estimation of recruitment and attrition rates; and evaluation of patient perceptions about the interventions from follow-up surveys. We will also compare medication adherence by intervention arm after enrollment. We will adjust these analyses for BMI, age, gender, race, forgetfulness (from the baseline and follow-up survey), and diabetic status. In addition, we will track BP measurements from the EMR that are obtained through usual care as well as during PCP visits, MPM messaging, phone calls; hospitalizations; and medication changes for up to 8 months after the study intervention using similar models. Lastly we will analyze diastolic blood pressure at the 4-month visit, adjusting for the initial diastolic blood pressure.

Study Design

1.1 Design

This is a prospective, randomized control trial: 150 patients will be randomized into one of three intervention arms: A) remote monitoring of blood pressure and medication adherence; B) remote monitoring of blood pressure and medication adherence with feedback to social support; and C) usual care. Participants will be randomized in a 2:2:1 ratio to the three study arms in variable blocks of 5 and 10 using a computer-generated randomization algorithm. The investigators will be blinded to the randomization assignment. The research coordinator and research assistants will be unblinded. The blind may be broken in the case of an emergency.

1.2 Consent Process

We are requesting a waiver of the requirement to document consent and HIPAA authorization with a signature for participants enrolled into this study since we believe the research presents no more than minimal risk of harm to subjects and involves no procedures for which written consent is normally required outside of the research context. A majority of participants being enrolled in this study will be enrolled via a remote recruitment process, therefore we will read the IRB approved Consent/HIPAA script over the phone to each patient and ask them to provide verbal consent and verbal HIPAA authorization for

use of their data in the study. Once a patient provides verbal consent, the research coordinator will select this option on the Consent/HIPAA screen on the participant dashboard that was just created for participation on the WTH health web platform for this study. At this point, the participant is randomized into 1 of the 3 study groups. After randomization, the coordinator will inform the participant of their assignment and read the instructions for their study group. A copy of the consent form will also be mailed to the participant's home address. A copy of the Consent/HIPAA document will also be included in the recruitment mailings sent to all patients before being called by the study team. While we do ask that participants come for an in-person end of study visit, it is likely that due to attrition many will not complete the final visit. As is such, we believe it impracticable to collect the signed consent form at this time, as we will end up with an incomplete record of written consent. Therefore, a waiver of the requirement to document consent and HIPAA authorization with a signature is requested.

Study Duration

We expect recruitment to take approximately 3 months. The intervention will be 4 months from the date of consent, and we will need an additional 3 months of follow-up, passively monitoring visit utilization and blood pressure from the electronic medical record. The CRC will also reach out to the feedback partner of patients in the social support arm. Including data analysis and manuscript preparation, total study duration should not exceed 12 months.

Resources Necessary for Human Research Protections

Shivan Mehta is the PI of this study. He is a gastroenterologist and the Associate Chief Innovation Officer at Penn Medicine. He is also an assistant professor of medicine at the Perelman School of Medicine, senior fellow at the Leonard Davis Institute of Health Economics, and affiliated faculty at the Center for Health Incentives and Behavioral Economics, all at the University of Pennsylvania All members of the research team have completed CITI human subjects research training. The Research Coordinator will provide thorough education and training to the Research Assistants to ensure that they are well-prepared to carry out the duties in their job descriptions. Additionally, the Research Coordinator will audit 10% of the electronic medical record reviews that the Research Assistants complete, in order to check for compliance. Detailed Standard Operating Procedure documents for the project will be accessible to all members of the research team, which will keep research staff informed about the protocol and their related duties. There are adequate facilities to conduct the research; all research staff have adequate office space on the UPenn campus.

Target Population

Eligibility Criteria: The study population includes patients 18-75 who have received care at the Penn Family Care practice, who've been diagnosed with HTN and have had at least 2 visits within the past year with BP readings that exceed HTN guidelines.

Subjects Enrolled by Penn Researchers

150

Subjects Enrolled by Collaborating Researchers
0

Accrual
Assuming a 5.3mmHg standard deviation of SBP (given variability of BP over time) and a two-sided significance level of 0.017 (to accommodate three pairwise comparisons), our sample size of 60 in each intervention arm and 30 in the control arm provides 80% power to detect a difference in SBP reduction of 3.75 mmHg between either intervention and usual care, and a difference in SBP reduction of 3.1 mmHg between the remote monitoring and social influcen arms. A preliminary data extra shows about 2300 uncontrolled hypertensive patients in Penn family Care, which should provide a sufficient population for enrollment.

Key Inclusion Criteria
1. Between 18 and 75 years old with HTN (ICD-10 code I10)
2. Has had at least two office visits at Penn Family Care (PFC) within the past 12 months (at time of chart review), with at least two of the visits with BP readings exceeding HTN guidelines, including the last visit (150/90 or 140/90 if ages 21-59 yrs with CKD or diabetes).
3. Must have a cellular phone with texting capabilities
4. Must be prescribed at least one medication for hypertension

Key Exclusion Criteria
1. Has metastatic (Stage IV) blood or solid tumor cancer
2. Has end stage renal disease
3. Has congestive heart failure
4. Has dementia
5. Has liver cirrhosis

Vulnerable Populations
No vulnerable Populations are included in the research study.

Populations Vulnerable to Undue Influence or Coercion
We are not specifically targeting any vulnerable populations.

Subject Recruitment
Participants in this study will be identified via electronic medical records review by our study staff. The study staff will work with UPHS to create a filter for the electronic medical record system that will generate a monthly list of patients who may meet the study criteria. A study coordinator will review the list of patients to determine eligibility. If patients meet the minimal requirements they will be added to the study screening data base and contacted via recruitment letter and up to 3 follow-up phone calls. If participants are interested, the coordinator will complete an intake form and consent via the remote enrollment process over the phone. The coordinator will enter patient information directly into the Way to Health web portal for this study. After providing consent, the patient will be randomized into 1 of the 3 study arms and will be read the script for that arm.

Subject Compensation
Subjects will receive a $25 check for their initial enrollment and completion of the baseline survey. Subjects will receive an additional $50 check after completing the final, end-of-study in person office visit and survey.

Procedures
Recruitment: The study staff will work with UPHS and the Penn Data Store to create a filter for the electronic medical record system that will generate a monthly list of patients who may meet our study criteria. A study coordinator will review the list of patients to determine eligibility. If patients meet the minimal requirements they will be added to the study screening database and will be mailed a recruitment letter and informational brochure, followed by up to three recruitment calls. During the recruitment phone call, a coordinator will ask the patient if they are interested in answering screening questions to confirm they are eligible to participate in this research study. If they are eligible, the coordinator will review the verbal consent/HIPAA script to the patient over the phone and obtain verbal consent and HIPAA authorization.

Enrollment: Enrollment will occur on the Way to Health platform for this study and patient information will be directly entered into the Way to Health website to create a participant study account. The patient will be mailed a copy of the consent document. After confirming consent to participate, the coordinator begins entering basic demographic information for the patient into the WTH site, including the name and contact information for a social support partner. All enrolled patients will receive a $25 incentive for their initial participation in the study. The research coordinator will collect the SSN of all study participants to enter into the W-9 webpage on the Way to Health web portal for this study to facilitate participant incentive payment. Providing SSN is not mandatory for enrollment but it is mandatory for receiving payments. The coordinator will ask the participant to provide details about medications they have been prescribed. After a patient enrolls, a message will be sent to their PCP via EPIC to notify their physician that their patient is enrolled in our study. Patients will be made aware of this message to their PCP in both the consent and enrollment process.

Randomization: The coordinator will use the Way to Health web platform to randomize patients into this study. Patients will be randomized into 1 of the 3 study arms (ratio 2:2:1) in variable blocks of 5 and 10. After randomization occurs, the study coordinator will inform the patient of the randomization group they have been assigned into. Arms A and B will receive a blood pressure cuff and bidirectional text messaging regarding blood pressure and medication adherence. Arm B will also include a social support partner to participate. Arm C will receive usual care. After randomization occurs, the study coordinator will inform the patient of the randomization group they have been assigned into. Those patients assigned to Arms A and B (remote monitoring) will be shipped the blood pressure cuff with instructions for use and a number to call us with questions. If we do not receive blood pressure readings from the patient 5 days after the patient received the package, a coordinator will call the patient to make sure the device is working properly. The coordinator will also reach out to the feedback partner in the social support arm to include in Way to Health.

Follow-Up Visit: During the enrollment call the research coordinator will assign the patient a date for their follow-up blood pressure measurement at the end of the 4 month intervention. The coordinator will assign the follow-up date in the hopes that using an opt-out method (assign the follow-up date to the patients) rather than an opt-in method (allow the patient to select their follow-up date) will increase attendance. However, the patient can change their follow-up date at any time. The intervention of the study will be 4

months from date of consent, and an additional 3 months of follow-up, passively monitoring visit utilization and blood pressure in the electronic medical record. At the conclusion of the intervention, participants will receive an additional $50 to complete an in-person blood pressure measurement and post-study survey. Patients will receive a letter one month prior to the follow-up appointment, and a text 2 weeks prior and 3 days prior to the appointment as a reminder of their appointment. The messages will be a reminder of the appointment date, time and location. Patients will also be instructed to call us if they would like to change their appointment. Information about BP measurements will be recorded in EPIC and message will be sent to the PCP with the research BP measurement included. When patients attend their follow-up measurement they will also be asked to complete a short follow-up survey. If a patient does not attend their in-person follow-up measurement a coordinator will call the patient to complete the survey over the phone.

Support Partners: During participant eligibility screening, potential participants will be asked for up to three friends or family members (in order) who'd be willing to support them through this study. They will be asked to provide that person's name and cell phone number (text capable cell phone is required), and to provide their permission to contact this partner, should the participant be eligible, consent, and be randomized to the Social Support arm of the study. Support partners will be contacted once a patient has been assigned to the social support arm. Details of their role will be provided, including that they will be texted once per week with an update on their participant's adherence to blood pressure monitoring and medications. They will be encouraged to support their participant in consistently monitoring their BP at least 3 times each week, and taking their medication(s) daily. Partners will be able to opt-out of a support message sent from the study to the participant on their behalf either congratulating them on their success or encouraging them to do better the following week. Once they agree, support partners will be enrolled directly via the Way to Health platform (a secure site) with name and cell phone number. If a participant is enrolled in the Social Support arm of the study and their identified partner declines participation, we will contact the next person on the list.

Analysis Plan

1.1 Power and Sample Size

Assuming 5.3mmHg standard deviation of SBP (given variability of BP over time) and a two-sided significance level of 0.017 (to accommodate the three pairwise comparisons), our sample size of 60 in each intervention arm and 30 in the control arm provides 80% power to detect a difference in SBP reduction of 3.75 mmHg between either intervention and usual care, and a difference in SBP reduction of 3.1 mmHg between the remote monitoring and social influence arms. A preliminary data extraction shows about 2300 patients uncontrolled hypertensive patients in PFC, which should provide a sufficient population for enrollment.

1.2 Data analysis

As a randomized controlled trial, we will conduct intention to treat analysis. For patients with missing data, we will first include blood pressure readings from the EMR and then conduct multiple imputations.
The primary outcome will relate the systolic blood pressure during the 4 month visit to intervention arm, adjusting for the initial systolic blood pressure by including baseline measures in the model. For patients with missing data who do not complete the 4 month in-person blood pressure reading, we will conduct multiple imputation of the SBP values at 4 months as a function of baseline variables, including intervention arm.

Secondary analyses include modeling of normotensive status using logistic regression; estimation of recruitment and attrition rates; and evaluation of patient perceptions about the interventions from follow-up surveys. We will also compare medication adherence by intervention arm after enrollment. We will adjust these analyses for BMI, age, gender, race, forgetfulness (from the baseline and follow-up survey), and diabetic status. In addition, we will track BP measurements from the EMR that are obtained through usual care as well as during PCP visits, MPM messaging, phone calls; hospitalizations; and medication changes for up to 8 months after the study intervention using similar models. Lastly we will analyze diastolic blood pressure at the 4-month visit, adjusting for the initial diastolic blood pressure.

Data Confidentiality

Paper-based records will be kept in a secure location and only be accessible to personnel involved in the study. Computer-based files will only be made available to personnel involved in the study through the use of access privileges and passwords. Prior to access to any study-related information, personnel will be required to sign statements agreeing to protect the security and confidentiality of identifiable information. Wherever feasible, identifiers will be removed from study-related information. Precautions are in place to ensure the data is secure by using passwords and encryption, because the research involves web-based surveys.

Subject Confidentiality

Information about study subjects will be kept confidential and managed according to the requirements of the Health Insurance Portability and Accountability Act of 1996 (HIPAA). All PHI will be maintained on UPHS servers. Source documents are maintained in PennChart. No source documents will be printed or maintained in paper form at the study site. Data from PennChart will be recorded in Penn Medicine's REDCap system. The investigator and study team (which includes the research coordinator, and research assistants) will have access to PHI within PennChart and REDCap. We will label all PHI within REDCap as identifiable information so that de-identified exports are possible. All reports that include identifiable information will be stored on the Innovation Center secure drive, maintained behind the UPHS firewall. Once data analysis and manuscripts have been published, the databases will be removed from REDCap and the data will be de-identified on the secure drive. This de- identified dataset will be stored for up to five years after analysis is complete and manuscripts have been published. Once analysis is completed and any manuscripts are published, we will retain PHI no longer than seven years in accordance with government regulations, applicable policies, and institutional requirements.

Database Security/Protection Against Risk

Information about study subjects will be kept confidential and managed according to the requirements of the Health Insurance Portability and Accountability Act of 1996 (HIPAA). All PHI will be maintained on UPHS servers. Source documents are maintained in PennChart. No source documents will be printed or maintained in paper form at the study site. To assure that patient, physician and other informant confidentiality is preserved, individual identifiers (such as name and medical record number/physician billing identifier) are stored in a single password protected system that is accessible only to study research, analysis and IT staff. This system is hosted on site at The University of Pennsylvania (UPenn) and is protected by a secure firewall. Once a participant is in this system, they will be given a unique study identification number (ID). Any datasets and computer files that leave the firewall will be stripped of all identifiers and individuals will be referred to by their study ID. The study ID will also be used on all analytical files.

The initial patient information collected for screening and recruitment will consist of name, address, phone number, demographic information such as race and income, and medication adherence practices. As our patient population is patients in the UPHS health system, the majority of this information will come from Electronic Chart reviews.

Sensitive Research Information

This Research does not involve collection of sensitive information about the subjects that should be excluded from the electronic medical record.

Subject Privacy

We will only interact with the subsample of subjects with which we plan to enroll in the study. With these subjects, we will conduct phone calls in a private area. When we call subjects, we will confirm the identify before beginning the recruitment process. When conducting the in-person 4-month visit, we will conduct blood pressure measurements and surveys in a private office location, with only one subject at a time.

Data Disclosure

Blood pressure measurements will be disclosed to the subject's primary care physician if they reach or surpass a pre-established threshold determined to be an immediate danger to the subject's health.

Protected Health Information/Data Protection

- Name
- Street address, city, county, precinct, zip code, and equivalent geocodes
- All elements of dates (except year) for dates directly related to an individual and all ages over 89
- Telephone and fax numbers
- Electronic mail addresses
- Medical record numbers
- Health Plan ID numbers

Consent Process

<u>1.1 Overview</u>

We are requesting a waiver of the requirement to document consent and HIPAA authorization with a signature for participants enrolled into this study since we believe the research presents no more than minimal risk of harm to subjects and involves no procedures for which written consent is normally required outside of the research context. A majority of participants being enrolled in this study will be enrolled via a remote recruitment process, therefore we will read the IRB approved Consent/HIPAA script over the phone to each patient and ask them to provide verbal consent and verbal HIPAA authorization for use of their data in the study. Once a patient provides verbal consent, the research coordinator will select this option on the Consent/HIPAA screen on the participant dashboard that was just created for participation on the WTH health web platform for this study. At this point, the participant is randomized into 1 of the 3 study groups. After randomization the coordinator will inform the participant of their assignment and read the instructions for their study group. A copy of the consent form will also be mailed to the participant's home address. A copy of the Consent/HIPAA document will also be included in the recruitment mailings sent to all patients before being called by the study team. While we do ask that participants come for an in-person end of study visit, it is likely that due to attrition many will not

complete the final visit. As is such, we believe it impracticable to collect the signed consent form at this time, as we will end up with an incomplete record of written consent. Therefore, a waiver of the requirement to document consent and HIPAA authorization with a signature is requested.

1.2 Children and Adolescents
Not applicable

1.3 Adult Subjects Not Competent to Give Consent
Waiver of consent is being requested.

Waiver of Consent
1.1 Minimal Risk
The research presents no more than minimal risk of harm to subjects and involves no procedures for which written consent is normally required outside of the research context.

1.2 Impact on Subjects Rights and Welfare
N/A

1.3 Waiver Essential to Research
N/A

1.4 Written Statement of Research
A copy of the Consent/HIPAA document will be included in the recruitment mailings sent to all patients before being called by the study team. After offering verbal consent to participate in the study, copy of the consent form will be mailed to the participant's home address.

Potential Study Risks
As this study does not involve any medical decision making and only tests the use of social behavioral approaches to encouraging patients to use evidence-based treatments that their providers have prescribed to treat high blood pressure, we consider this study minimal risk. The primary risk would be from a breach of confidentiality involving medical records reviews and monitoring of hypertension medication and blood pressure adherence with text messaging which will be maintained on the Way to Health platform. This risk has been mitigated by extensive privacy protection protocols, a highly secure data storage system, and a plan to remove identifiers from the data wherever possible. In addition, all personnel will be held to high standards of upholding confidentiality and safeguarding patient privacy.

Potential Study Benefits
The immediate benefits of this study for participants may include an improvement in adherence to medications that have been proven to be effective in improving patient outcomes. It is possible that the benefits for many participants will be minimal. However, as mentioned, we believe the risks are also minimal. Knowledge gained from the study will assist in development of interventions in other high risk patient populations in which non-adherence rates are high. The potential public health impact of a successful intervention to improve adherence to medications is great and could reduce the number of deaths from health related outcomes in the United States each year.

Data and Safety Monitoring

The study PI will be responsible for monitoring the data and safety of the study, and ensuring the ongoing quality and integrity of the research study. The investigator will permit study-related monitoring, audits and inspections by government regulatory authorities and University compliance officers.

Risk/Benefit Assessment

Medication adherence is an important factor contributing to poor control of a number of chronic diseases, including hypertension, hyperlipidemia, coronary artery disease, and diabetes. Many studies show that adherence to simple medication regimens is suboptimal with less than half of patients taking medications in the year after initial prescription. This study is designed to test an intervention that incorporates many components that have previously demonstrated improvement in past studies. We believe the combination of these approaches in this study will provide the research and public health communities with important information that can lead to broad generalizability in treating people at risk for the above mentioned chronic diseases and death nationally, as these types of interventions could be set up by healthcare systems to be broadly utilized.

Final Protocol

****New changes from initial protocol notated in bold, parts removed from initial protocol notated in strikethrough**

Abstract
A 3-arm randomized pilot trial aimed at comparing the effectiveness of different approaches to improving blood pressure (BP) control: A) remote monitoring of BP and medication adherence; B) remote monitoring of BP and medication adherence with feedback to social support; and C) usual care. The targeted population is patients aged 18-75 within the Penn Family Care practice with at least 2 visits within the prior 12 months with blood pressure readings exceeding recommended HTN guidelines.

Study Instruments
All subjects will complete a baseline and post-intervention survey. The baseline survey will collect basic demographics and baseline medication adherence and blood pressure monitoring frequency. The post-intervention survey will collect~~ion~~ similar adherence and monitoring information, as well as qualitative data regarding patient perceptions about the interventions. **Provider interviews following the intervention period will collect clinician and care team perceptions about the interventions as well as feedback for improvement of future iterations.**

Group Modifications
For subjects in the Control (usual care) arm of the study, the subjects will not receive text reminders to monitor their blood pressure or take their medication(s). For subjects in the Remote Monitoring plus Social Support (RM+SS) arm of the study, the subjects will receive additional feedback from a designated support partner.

Administration of Surveys
All subjects will complete a baseline and post-intervention survey. The baseline survey will collect basic demographics and baseline medication adherence and blood pressure monitoring frequency and be conducted over the phone after the participant has been consented. The post-intervention survey will collect similar adherence and monitoring information, as well as qualitative data regarding patient perceptions about the interventions. The post-intervention survey will be completed at the in-person 4 month visit. These surveys should take no more than 15 minutes to complete. Demographic and socio-economic questions are modified from demographic questions on the Behavioral Risk Factor Surveillance System survey, administered by the Centers for Disease Control and Prevention. **Provider interviews following the intervention period will collect clinician and care team perceptions about the interventions as well as feedback for improvement of future iterations. Provider interviews should take no more than 20-25 minutes to complete.**

Objectives
<u>1.4 Objectives</u>
We plan to leverage the Way to Health (WTH) platform to develop and evaluate a new HTN service-delivery model that is integrated with routine clinical care at Penn Family Care (PFC), an academic family medicine practice in West Philadelphia. Among patients with poorly controlled HTN:

3. We will assess the effects of monitoring blood pressure and medication adherence with feedback to the patient and to the clinical practice, as needed if out of control.
4. We will also compare the impact of providing feedback to a social support partner with what

we call facilitated cheerleading.

1.5 <u>Primary Outcome Variable</u>
The primary outcome is systolic blood pressure during the 4-month study visit by intervention arm, adjusting for the initial systolic blood pressure. For patients with missing data who do not complete the 4 month in-person blood pressure reading, we will conduct multiple imputation of the SBP values at 4 months as a function of baseline variables, including intervention arm.

1.6 <u>Secondary Outcome Variable(s)</u>
Secondary outcomes include the percent of patients that are normotensive during the 4 month study visit, estimation of recruitment and attrition rates, **and evaluation of** patient perceptions about the intervention **from surveys**. We will also compare medication adherence **by intervention arm** after enrollment, adjusted for BMI, age, gender, race, forgetfulness (from the baseline and follow-up surveys), and diabetic status. In addition, we will track BP measurements from the EMR that are obtained through usual care as well as during PCP visits, MPM messaging, and phone calls; **number of office visits** and hospitalizations; and medication changes for up to ~~9~~ ~~3~~**8** months after the study intervention using similar models. We will analyze diastolic blood pressure at the 4-month visit, adjusting for the initial diastolic blood pressure. ~~Lastly, w~~**We will measure and compare medication and blood pressure adherence between the two intervention arms, allowing us a more proximal metric to see if social influence makes a difference on adherence with texting. Lastly, we will evaluate qualitative data regarding provider experience with the intervention as well as feedback about how the intervention could be improved for future iterations. This data will be supplemented with information gathered from a review of provider actions following monitoring encounters.**

Background
<u>1.1 Program Goals</u>
Hypertension (HTN) affects about 30% of US adults, and effective treatment that reduces long-term risk is available, but only about half of adults maintain good control. HTN control requires diagnosis, initiation of treatment, adherence to medications, and titration of medications. The traditional visit-based primary care model is inadequate to address management needs, given the limited visit time and lack of insight into the behavior of patients outside of the clinic setting.

Substantial literature has shown the benefit of remote monitoring interventions in controlling HTN, but the effects are not typically sustained and traditional primary care practices may not have the infrastructure to respond to remote monitoring data. There is an opportunity to develop automated approaches to monitoring blood pressure and medication adherence by leveraging principles of behavioral economics and clinical practice re-design. Our focus is on self-monitoring of blood pressure and medication adherence using bi-directional text messaging.

In addition to providing feedback to patients, we will also engage a friend or family member identified by the patient who has agreed to act as a feedback partner. Those with active social support typically have better clinical outcomes, and the feedback partner could serve as a witness to non-adherence as well as a cheerleader. Despite considerable enthusiasm for the concept that social support can be used to advance medication adherence, there is currently no evidence that this provides benefit beyond individual feedback. The tepid results from those few studies may be explained by limited participation of the feedback partner or an insufficient dose of cheerleading. There may be greater impact if we make it easier for them to communicate with the patient to provide motivation **through the use of opt-out messaging.**

We plan to leverage the Way to Health (WTH) platform to develop and evaluate a new HTN serve delivery model that is integrated with routine clinical care at Penn Family Care (PFC), an academic family medicine practice in West Philadelphia. Among patients with poorly controlled HTN, we will assess the effects of monitoring blood pressure and medication adherence with feedback to the patient and to the clinical practice, as needed if out of control. We will also compare the impact of providing feedback to a social support partner with what we call facilitated cheerleading.

~~Some health systems have employed a proactive approach to identifying eligible patients and encouraging participation in CRC screening through direct outreach, thereby eliminating barriers to screening such as the in-office visit. Kaiser Permanente Northern California is one such example that improved their screening rates to over 80% through mailed FIT outreach to eligible patients. The opportunity exists to extend this model to other health systems, such as UPHS, to engage in organized direct outreach with mailed FIT in order to enhance CRC screening rates.~~

~~In this study, we will be using population-based outreach screening to better understand mailed CRC screening uptake. By evaluating the effectiveness of opt out versus opt in mailed outreach screening, we will enhance the public health capacity, and efficiency, to increase CRC screening uptake and reduce preventable death from this disease. Default settings (such as opt out) have been effective in producing desired behavior actions in a variety of economic and clinical contexts, including employee enrollment in retirement savings, organ donation, subject enrollment in research studies and breast screening. However, there is a lack of evidence for use in CRC screening. Moreover, by furthering our understanding of patient-level facilitators and barriers to completing mailed FIT, we hope to support the need for further research to improve the delivery of CRC screening and screening uptake in diverse population groups.~~

Statistical Considerations

1.1 Power and sample size

Assuming 5.3mmHg standard deviation of SBP (given variability of BP over time) and a two-sided significance level of 0.017 (to accommodate the three pairwise comparisons), our sample size of 60 in each intervention arm and 30 in the control arm provides 80% power to detect a difference in SBP reduction of 3.75 mmHg between either intervention and usual care, and a difference in SBP reduction of 3.1 mmHg between the remote monitoring and social influence arms. A preliminary data extraction shows about 2300 patients uncontrolled hypertensive patients in PFC, which should provide a sufficient population for enrollment. **However, based on additional clinical information obtained after the study was initiated, we estimate a standard deviation for change in SBP of 20mmHg, larger than our initial estimate. Thus, we increased our accrual target to 100 in each intervention arm and 50 in the control arm, which provides 80% power to detect a difference in SBP reduction of 11mmHg between either intervention and usual care, and a difference in SBP reduction of 9mmHg between the remote monitoring and social influence arms.**

1.2 Data analysis

As **is standard in** a randomized controlled trial, ~~we will conduct~~**our primary analysis will follow the** intention to treat **approach, in which participants are analyzed according to their assigned treatment regardless of compliance. analysis.** For patients with missing **blood pressure** data, we will first include ~~blood pressure~~ readings **available** from the **electronic health record (**EH~~M~~R**) occurring from 90-150 days from the participant's start in the intervention,** and then conduct multiple imputation **using all available baseline covariates**.

The primary ~~outcome~~ **analysis** will relate the systolic blood pressure ~~during~~**at** the 4 month visit to **assigned** intervention arm, adjusting for the initial systolic blood pressure by including **the** baseline measures in the model. ~~For patients with missing data who do not complete the 4 month in-person blood pressure reading, we will conduct multiple imputation of the SBP values at 4 months as a function of baseline variables, including intervention arm.~~

Secondary analyses include ~~modeling~~ **analyzing achievement** of normotens**ion**~~ive status~~**by study arm** using logistic regression **and repeating the analyses for systolic blood pressure with diastolic blood pressure at the 4-month visit. We will** estimat~~eion of~~ recruitment and attrition rates, and evaluat~~eion of~~ patient perceptions about the interventions from follow-up surveys. We will also compare medication adherence **and blood pressure monitoring adherence** by intervention arm after enrollment. We will adjust these analyses for BMI, age, gender, race, forgetfulness (from the baseline and follow-up survey), and diabetic status. In addition, we will track BP measurements **by arm** from the **(**E**H**~~M~~R**)** that are obtained through usual care **for up to 8 months after the study intervention. We will** ~~as well as during~~**also evaluate** PCP visits, MPM messaging, phone calls, hospitalizations, and medication changes. ~~for up to 8 months after the study intervention using similar models. Lastly we will analyze diastolic blood pressure at the 4-month visit, adjusting for the initial diastolic blood pressure.~~**We will repeat all analyses by sub-group related to time of enrollment (round 1 and round 2). Finally, we will perform a thematic evaluation of provider perceptions about the interventions from information gathered during follow-up telephone interviews as well as process data collected from a review of provider actions following monitoring encounters.**

Study Design

<u>1.1 Design</u>

This is a prospective, randomized controll**ed** trial: ~~150~~ **250** patients will be randomized into one of three intervention arms: A) remote monitoring of blood pressure and medication adherence; B) remote monitoring of blood pressure and medication adherence with feedback to social support; and C) usual care. Participants will be randomized in a 2:2:1 ratio to the three study arms in variable blocks of 5 and 10 using a computer-generated randomization algorithm. The investigators will be blinded to the randomization assignment. The research coordinator and research assistants will be unblinded. The blind may be broken in the case of an emergency.

<u>1.2 Consent Process</u>

We are requesting a waiver of the requirement to document consent and HIPAA authorization with a signature for participants enrolled into this study since we believe the research presents no more than minimal risk of harm to subjects and involves no procedures for which written consent is normally required outside of the research context. A majority of participants being enrolled in this study will be enrolled via a remote recruitment process, therefore we will read the IRB approved Consent/HIPAA script over the phone to each patient and ask them to provide verbal consent and verbal HIPAA authorization for use of their data in the study. Once a patient provides verbal consent, the research coordinator will select this option on the Consent/HIPAA screen on the participant dashboard that was just created for participation on the WTH health web platform for this study. At this point, the participant is randomized into 1 of the 3 study groups. After randomization, the coordinator will inform the participant of their assignment and read the instructions for their study group. A copy of the consent form will also be mailed to the participant's home address. A copy of the Consent/HIPAA document will also be included in the recruitment mailings sent to all patients before being called by the study team. While we do ask that

participants come for an in-person end of study visit, it is likely that due to attrition many will not complete the final visit. As is such, we believe it impracticable to collect the signed consent form at this time, as we will end up with an incomplete record of written consent. Therefore, a waiver of the requirement to document consent and HIPAA authorization with a signature is requested.

Study Duration
We expect recruitment to take approximately 3 months. The intervention will be 4 months from the date of consent, and we will need an additional ~~3~~ **8** months of follow-up, passively monitoring visit utilization and blood pressure from the electronic medical record. The CRC will also reach out to the feedback partner of patients in the social support arm. Including data analysis and manuscript preparation, total study duration should not exceed 12 months.

Resources Necessary for Human Research Protections
Shivan Mehta is the PI of this study. He is a gastroenterologist and the Associate Chief Innovation Officer at Penn Medicine. He is also an assistant professor of medicine at the Perelman School of Medicine, senior fellow at the Leonard Davis Institute of Health Economics, and affiliated faculty at the Center for Health Incentives and Behavioral Economics, all at the University of Pennsylvania All members of the research team have completed CITI human subjects research training. The Research Coordinator will provide thorough education and training to the Research Assistants to ensure that they are well-prepared to carry out the duties in their job descriptions. Additionally, the Research Coordinator will audit 10% of the electronic medical record reviews that the Research Assistants complete, in order to check for compliance. Detailed Standard Operating Procedure documents for the project will be accessible to all members of the research team, which will keep research staff informed about the protocol and their related duties. There are adequate facilities to conduct the research; all research staff have adequate office space on the UPenn campus.

Target Population
Eligibility Criteria: The study population includes patients 18-75 who have received care at the Penn Family Care practice, who've been diagnosed with HTN and have had at least 2 visits within the past **two** year**s** with BP readings that exceed HTN guidelines.

Subjects Enrolled by Penn Researchers
~~150~~ **250**

Subjects Enrolled by Collaborating Researchers
0

Accrual
Assuming a 5.3mmHg standard deviation of SBP (given variability of BP over time) and a two-sided significance level of 0.017 (to accommodate three pairwise comparisons), our sample size of 60 in each intervention arm and 30 in the control arm provides 80% power to detect a difference in SBP reduction of 3.75 mmHg between either intervention and usual care, and a difference in SBP reduction of 3.1 mmHg between the remote monitoring and social ~~influcen~~**influence** arms. A preliminary data extra**ction** shows about 2300 uncontrolled hypertensive patients in Penn family Care, which should provide a sufficient population for enrollment. **However, based on additional clinical information obtained after the study**

was initiated, we estimate a standard deviation for change in SBP of 20mmHg, larger than our initial estimate. Thus, we increased our accrual target to 100 in each intervention arm and 50 in the control arm, which provides 80% power to detect a difference in SBP reduction of 11mmHg between either intervention and usual care, and a difference in SBP reduction of 9mmHg between the remote monitoring and social influence arms.

Key Inclusion Criteria
1. Between 18 and 75 years old with HTN (ICD-10 code I10)
2. Has had at least two office visits at Penn Family Care (PFC) within the past ~~12~~ **24** months (at time of chart review), with at least two of the visits with BP readings exceeding HTN guidelines, including the last visit (150/90 or 140/90 if ages 21-59 yrs ~~with~~ **or has** CKD or diabetes).
3. Must have a cellular phone with texting capabilities
4. Must be prescribed at least one medication for hypertension

Key Exclusion Criteria
1. Has metastatic (Stage IV) blood or solid tumor cancer
2. Has end stage renal disease
3. Has congestive heart failure
4. Has dementia
~~5. Has liver cirrhosis~~
5. Has a BMI >=50

Vulnerable Populations
No vulnerable Populations are included in the research study.

Populations Vulnerable to Undue Influence or Coercion
We are not specifically targeting any vulnerable populations.

Subject Recruitment
Participants in this study will be identified via electronic medical records review by our study staff. The study staff will work with UPHS to create a filter for the electronic medical record system that will generate a ~~monthly~~ list of patients who may meet the study criteria. A study coordinator will review the list of patients to determine eligibility. If patients meet the minimal requirements they will be added to the study screening data base and contacted via recruitment letter and up to ~~3~~ **5** follow-up phone calls. If participants are interested, the coordinator will complete an intake form and consent via the remote enrollment process over the phone. The coordinator will enter patient information directly into the Way to Health web portal for this study. After providing consent, the patient will be randomized into 1 of the 3 study arms and will be read the script for that arm.

Subject Compensation
~~Subjects~~ **Participants** will receive a $25 check for their initial enrollment and completion of the baseline survey. ~~Subjects~~ **Participants** will receive an additional $50 check after completing the final, end-of-study in person office visit and survey**. Providers will be given a $25 Amazon gift card for participating in the**

follow-up telephone interviews.

Procedures

Recruitment: The study staff will work with UPHS and the Penn Data Store to create a filter for the electronic medical record system that will generate a ~~monthly~~ list of patients who may meet our study criteria. A study coordinator will review the list of patients to determine eligibility. If patients meet the minimal requirements they will be added to the study screening database and will be mailed a recruitment letter and informational brochure, followed by up to ~~three~~ **5** recruitment calls. During the recruitment phone call, a coordinator will ask the patient if they are interested in answering screening questions to confirm they are eligible to participate in this research study. If they are eligible, the coordinator will review the verbal consent/HIPAA script to the patient over the phone and obtain verbal consent and HIPAA authorization.

Enrollment: Enrollment will occur on the Way to Health platform for this study and patient information will be directly entered into the Way to Health website to create a participant study account. The patient will be mailed a copy of the consent document. After confirming consent to participate, the coordinator begins entering basic demographic information for the patient into the WTH site, including the name and contact information for a social support partner. All enrolled patients will receive a $25 incentive for their initial participation in the study. The research coordinator will collect the SSN of all study participants to enter into the W-9 webpage on the Way to Health web portal for this study to facilitate participant incentive payment. Providing SSN is not mandatory for enrollment but it is mandatory for receiving payments. The coordinator will ask the participant to provide details about medications they have been prescribed. After a patient enrolls, a message will be sent to **a designated nursing pool and** their PCP via EPIC to notify their physician that their patient is enrolled in our study. Patients will be made aware of this message to their PCP in both the consent and enrollment process. **For patients in the intervention arms, this message will also include a pended procedure order authorizing patient submitted blood pressure measurements to be reviewed via a specially created flowsheet linking Way to Health to Epic.**

Randomization: The coordinator will use the Way to Health web platform to randomize patients into this study. Patients will be randomized into 1 of the 3 study arms (ratio 2:2:1) in variable blocks of 5 and 10. After randomization occurs, the study coordinator will inform the patient of the randomization group they have been assigned into. Arms ~~A~~ **1** and ~~B~~ **2** will receive a blood pressure cuff and bidirectional text messaging regarding blood pressure and medication adherence. Arm ~~B~~ **2** will also include a social support partner to participate. Arm ~~C~~**3** will receive usual care. After randomization occurs, the study coordinator will inform the patient of the randomization group they have been assigned into. Those patients assigned to Arms ~~A~~ **1** and ~~B~~ **2** (remote monitoring) will be shipped the blood pressure cuff with instructions for use and a number to call us with questions. If we do not receive blood pressure readings from the patient ~~5~~ **10** days after the patient received the package, a coordinator will call the patient to make sure the device is working properly. The coordinator will also reach out to the feedback partner in the social support arm to include in Way to Health.

Monitoring: Participants in Arms 1 and 2 will be asked to submit blood pressure measurements three times each week for the duration of the study. If at any point, 3 out of the last 10 reported BP measurements are elevated, the elevated measurements and any measurements between (up to 10, with a minimum of 5 if available), will be reported to the Nursing Pool ~~PCP~~ via a medical record encounter. This encounter will also reference the flowsheet so the care team may view all patient submitted measurements to date, if needed or desired. Information about self-reported medication

adherence will also be reported ~~to the PCP~~ at this time. Nursing pool staff will meet twice weekly with an assigned Nurse Practitioner (NP) to review all monitoring notification sin bulk, and make appropriate medication adjustments with follow-up with the patient as needed. The final encounter will be routed by the nursing pool to the PCP as an FYI. Measurements will be reported again in this same manner if they remain elevated and 3 weeks have elapsed since last reported.

Additionally, if a participant in Arm 1 or Arm 2 submits any single elevated blood pressure measurement (>=180 and/or >=110), this measurement along with information about self-reported medication adherence will be reported to the Nursing Pool ~~PCP~~ via a medical record encounter and the same procedure as outlined above will be followed for proper patient follow-up.

Follow-Up Visit: During the enrollment call the research coordinator will assign the patient a date for their follow-up blood pressure measurement at the end of the 4 month intervention. The coordinator will assign the follow-up date in the hopes that using an opt-out method (assign the follow-up date to the patients) rather than an opt-in method (allow the patient to select their follow-up date) will increase attendance. However, the patient can change their follow-up date at any time. The intervention of the study will be 4 months from date of consent, and an additional ~~3~~ **8** months of follow-up, passively monitoring visit utilization and blood pressure in the electronic medical record. At the conclusion of the intervention, participants will receive an additional $50 to complete an in-person blood pressure measurement and post-study survey. Patients will receive a letter one month prior to the follow-up appointment, and a text 2 weeks prior~~, and~~ 3 days prior**, and 1 day prior** to the appointment as a reminder of their appointment. The messages will be a reminder of the appointment date, time and location**, as well as of the incentive for completion of the visit**. Patients will also be instructed to call us if they would like to change their appointment. Information about BP measurements will be recorded in EPIC and message will be sent to the **Nursing Pool and** PCP with the research BP measurement included. When patients attend their follow-up measurement they will also be asked to complete a short follow-up survey. If a patient does not attend their in-person follow-up measurement, a coordinator will call the patient to **reschedule the visit and if that is not possible, to** complete the survey over the phone.

Support Partners: During participant eligibility screening, potential participants will be asked for up to three friends or family members (in order) who'd be willing to support them through this study. They will be asked to provide that person's name and cell phone number (text capable cell phone is required), and to provide their permission to contact this partner, should the participant be eligible, consent, and be randomized to the Social Support arm of the study. Support partners will be contacted once a patient has been assigned to the social support arm. Details of their role will be provided, including that they will be texted once per week with an update on their participant's adherence to blood pressure monitoring and medications. They will be encouraged to support their participant in consistently monitoring their BP at least 3 times each week, and taking their medication(s) daily. Partners will be able to opt-out of a support message sent from the study to the participant on their behalf either congratulating them on their success or encouraging them to do better the following week. Once they agree, support partners will be enrolled directly via the Way to Health platform (a secure site) with name and cell phone number. If a participant is enrolled in the Social Support arm of the study and their identified partner declines participation, we will contact the next person on the list.

Encounter Handling: Following completion of the intervention period, we will review and document clinician and care team actions after receiving a monitoring encounter from the study team. Information collected will include medication changes, telephone encounters with patients, office visits and nurse interactions.

Provider Interviews: Following completion of the intervention period, we will interview providers who had patients enrolled in the study regarding their perceptions regarding the intervention, as well as solicit feedback for improving future iterations. Telephone interviews are expected to last between 20-25 minutes. Providers will be offered a $25 Amazon gift card for their participation.

Analysis Plan

1.1 Power and Sample Size

Assuming 5.3mmHg standard deviation of SBP (given variability of BP over time) and a two-sided significance level of 0.017 (to accommodate the three pairwise comparisons), our sample size of 60 in each intervention arm and 30 in the control arm provides 80% power to detect a difference in SBP reduction of 3.75 mmHg between either intervention and usual care, and a difference in SBP reduction of 3.1 mmHg between the remote monitoring and social influence arms. A preliminary data extraction shows about 2300 patients uncontrolled hypertensive patients in PFC, which should provide a sufficient population for enrollment. **However, based on additional clinical information obtained after the study was initiated, we estimate a standard deviation for change in SBP of 20mmHg, larger than our initial estimate. Thus, we increased our accrual target to 100 in each intervention arm and 50 in the control arm, which provides 80% power to detect a difference in SBP reduction of 11mmHg between either intervention and usual care, and a difference in SBP reduction of 9mmHg between the remote monitoring and social influence arms.**

1.2 Data analysis

As **is standard in** a randomized controlled trial, ~~we will conduct~~**our primary analysis will follow the** intention to treat **approach, in which participants are analyzed according to their assigned treatment regardless of compliance. analysis.** For patients with missing **blood pressure** data, we will first include ~~blood pressure~~ readings **available** from the **electronic health record (**E**H**~~M~~R**) occurring from 90-150 days from the participant's start in the intervention,** and then conduct multiple imputation **using all available baseline covariates**.

The primary ~~outcome~~ **analysis** will relate the systolic blood pressure ~~during~~**at** the 4 month visit to **assigned** intervention arm, adjusting for the initial systolic blood pressure by including **the** baseline measures in the model. ~~For patients with missing data who do not complete the 4 month in-person blood pressure reading, we will conduct multiple imputation of the SBP values at 4 months as a function of baseline variables, including intervention arm.~~

Secondary analyses include ~~modeling~~ **analyzing achievement** of normotens**ion**~~ive status~~**by study arm** using logistic regression **and repeating the analyses for systolic blood pressure with diastolic blood pressure at the 4-month visit. We will** estimat**e**~~ion of~~ recruitment and attrition rates, and evaluat**e**~~ion of~~ patient perceptions about the interventions from follow-up surveys. We will also compare medication adherence **and blood pressure monitoring adherence** by intervention arm after enrollment. We will adjust these analyses for BMI, age, gender, race, forgetfulness (from the baseline and follow-up survey), and diabetic status. In addition, we will track BP measurements **by arm** from the **(**E**H**~~M~~R**)** that are obtained through usual care **for up to 8 months after the study intervention. We will** ~~as well as during~~**also evaluate** PCP visits, MPM messaging, phone calls, hospitalizations, and medication changes. ~~for up to 8 months after the study intervention using similar models. Lastly we will analyze diastolic blood pressure at the 4-month visit, adjusting for the initial diastolic blood pressure.~~**We will repeat all analyses by sub-group related to time of enrollment (round 1 and round 2). Finally, we will perform a thematic evaluation of**

provider perceptions about the interventions from information gathered during follow-up telephone interviews as well as process data collected from a review of provider actions following monitoring encounters.

Data Confidentiality

Paper-based records will be kept in a secure location and only be accessible to personnel involved in the study. Computer-based files will only be made available to personnel involved in the study through the use of access privileges and passwords. Prior to access to any study-related information, personnel will be required to sign statements agreeing to protect the security and confidentiality of identifiable information. Wherever feasible, identifiers will be removed from study-related information. Precautions are in place to ensure the data is secure by using passwords and encryption, because the research involves web-based surveys.

Subject Confidentiality

Information about study subjects will be kept confidential and managed according to the requirements of the Health Insurance Portability and Accountability Act of 1996 (HIPAA). All PHI will be maintained on UPHS servers. Source documents are maintained in PennChart. No source documents will be printed or maintained in paper form at the study site. Data from PennChart will be recorded in Penn Medicine's REDCap system. The investigator and study team (which includes the research coordinator, and research assistants) will have access to PHI within PennChart and REDCap. We will label all PHI within REDCap as identifiable information so that de-identified exports are possible. All reports that include identifiable information will be stored on the Innovation Center secure drive, maintained behind the UPHS firewall. Once data analysis and manuscripts have been published, the databases will be removed from REDCap and the data will be de-identified on the secure drive. This de- identified dataset will be stored for up to five years after analysis is complete and manuscripts have been published. Once analysis is completed and any manuscripts are published, we will retain PHI no longer than seven years in accordance with government regulations, applicable policies, and institutional requirements.

Database Security/Protection Against Risk

Information about study subjects will be kept confidential and managed according to the requirements of the Health Insurance Portability and Accountability Act of 1996 (HIPAA). All PHI will be maintained on UPHS servers. Source documents are maintained in PennChart. No source documents will be printed or maintained in paper form at the study site. To assure that patient, physician and other informant confidentiality is preserved, individual identifiers (such as name and medical record number/physician billing identifier) are stored in a single password protected system that is accessible only to study research, analysis and IT staff. This system is hosted on site at The University of Pennsylvania (UPenn) and is protected by a secure firewall. Once a participant is in this system, they will be given a unique study identification number (ID). Any datasets and computer files that leave the firewall will be stripped of all identifiers and individuals will be referred to by their study ID. The study ID will also be used on all analytical files.

The initial patient information collected for screening and recruitment will consist of name, address, phone number, demographic information such as race and income, and medication adherence practices. As our patient population is patients in the UPHS health system, the majority of this information will come

from Electronic Chart reviews.

Sensitive Research Information

This Research does not involve collection of sensitive information about the subjects that should be excluded from the electronic medical record.

Subject Privacy

We will only interact with the subsample of subjects with which we plan to enroll in the study. With these subjects, we will conduct phone calls in a private area. When we call subjects, we will confirm the identify before beginning the recruitment process. When conducting the in-person 4-month visit, we will conduct blood pressure measurements and surveys in a private office location, with only one subject at a time.

Data Disclosure

Blood pressure measurements will be disclosed to the subject's primary care physician if they reach or surpass a pre-established threshold determined to be an immediate danger to the subject's health.

Protected Health Information/Data Protection

- Name
- Street address, city, county, precinct, zip code, and equivalent geocodes
- All elements of dates (except year) for dates directly related to an individual and all ages over 89
- Telephone and fax numbers
- Electronic mail addresses
- Medical record numbers
- Health Plan ID numbers

Consent Process

1.1 Overview

We are requesting a waiver of the requirement to document consent and HIPAA authorization with a signature for participants enrolled into this study since we believe the research presents no more than minimal risk of harm to subjects and involves no procedures for which written consent is normally required outside of the research context. A majority of participants being enrolled in this study will be enrolled via a remote recruitment process, therefore we will read the IRB approved Consent/HIPAA script over the phone to each patient and ask them to provide verbal consent and verbal HIPAA authorization for use of their data in the study. Once a patient provides verbal consent, the research coordinator will select this option on the Consent/HIPAA screen on the participant dashboard that was just created for participation on the WTH health web platform for this study. At this point, the participant is randomized into 1 of the 3 study groups. After randomization the coordinator will inform the participant of their assignment and read the instructions for their study group. A copy of the consent form will also be mailed to the participant's home address. A copy of the Consent/HIPAA document will also be included in the recruitment mailings sent to all patients before being called by the study team. While we do ask that participants come for an in-person end of study visit, it is likely that due to attrition many will not complete the final visit. As is such, we believe it impracticable to collect the signed consent form at this

time, as we will end up with an incomplete record of written consent. Therefore, a waiver of the requirement to document consent and HIPAA authorization with a signature is requested.

1.2 Children and Adolescents
Not applicable

1.3 Adult Subjects Not Competent to Give Consent
Waiver of consent is being requested.

Waiver of Consent
1.1 Minimal Risk
The research presents no more than minimal risk of harm to subjects and involves no procedures for which written consent is normally required outside of the research context.

1.2 Impact on Subjects Rights and Welfare
N/A

1.3 Waiver Essential to Research
N/A

1.4 Written Statement of Research
A copy of the Consent/HIPAA document will be included in the recruitment mailings sent to all patients before being called by the study team. After offering verbal consent to participate in the study, copy of the consent form will be mailed to the participant's home address.

Potential Study Risks
As this study does not involve any medical decision making and only tests the use of social behavioral approaches to encouraging patients to use evidence-based treatments that their providers have prescribed to treat high blood pressure, we consider this study minimal risk. The primary risk would be from a breach of confidentiality involving medical records reviews and monitoring of hypertension medication and blood pressure adherence with text messaging which will be maintained on the Way to Health platform. This risk has been mitigated by extensive privacy protection protocols, a highly secure data storage system, and a plan to remove identifiers from the data wherever possible. In addition, all personnel will be held to high standards of upholding confidentiality and safeguarding patient privacy.

Potential Study Benefits
The immediate benefits of this study for participants may include an improvement in adherence to medications that have been proven to be effective in improving patient outcomes. It is possible that the benefits for many participants will be minimal. However, as mentioned, we believe the risks are also minimal. Knowledge gained from the study will assist in development of interventions in other high risk patient populations in which non-adherence rates are high. The potential public health impact of a successful intervention to improve adherence to medications is great and could reduce the number of deaths from health related outcomes in the United States each year.

Data and Safety Monitoring
The study PI will be responsible for monitoring the data and safety of the study, and ensuring the ongoing quality and integrity of the research study. The investigator will permit study-related monitoring, audits and inspections by government regulatory authorities and University compliance officers.

Risk/Benefit Assessment
Medication adherence is an important factor contributing to poor control of a number of chronic diseases, including hypertension, hyperlipidemia, coronary artery disease, and diabetes. Many studies show that adherence to simple medication regimens is suboptimal with less than half of patients taking medications in the year after initial prescription. This study is designed to test an intervention that incorporates many components that have previously demonstrated improvement in past studies. We believe the combination of these approaches in this study will provide the research and public health communities with important information that can lead to broad generalizability in treating people at risk for the above mentioned chronic diseases and death nationally, as these types of interventions could be set up by healthcare systems to be broadly utilized.

Summary of Protocol Changes Modifications LOG

Protocol: Remote Monitoring and Enhanced Social Support for Hypertension Management
University of Pennsylvania Principal Investigator: Shivan Mehta, MD

Date of Submission	Description of Modification	Rationale for Modification	Approval date
10/24/2017	Initial submission		11/6/16
11/30/2017	1) Updating study personnel<br>2) New patient outreach materials (brochure)<br>3) Updated Welcome Letter language<br>4) New non-compliance patient letter<br>5) Updated text messaging content<br>6) Updated outcomes<br>7) Updated protocol format	1) Additional staff to aid with testing, recruitment and monitoring<br>2) Informational brochure mailed ahead of recruitment phone call<br>3) Provides more detailed information about study participation to patient, including instruction for responding to study texts<br>4) Outreach for patients not reachable by phone if they have not been participating for 21+ days<br>5) Additions and edits to existing messaging for clarity and patient safety<br>6) Identified additional secondary outcomes for more thorough comparison of study groups<br>7) Updated format for ease in tracking changes through duration of study	12/27/17
02/06/2018	1) Updating study personnel<br>2) New/Updated patient materials	1) Remove 2 research assistants no longer working with study team<br>2) A. Welcome letters (2, updated) – provides additional language with more detailed information about high and low blood pressure readings. This language was not added to the control group welcome letter since they are not being monitored<br>2) B. Introductory letter (new) – letter mailed with informational brochure ahead of recruitment phone call	2/14/18
02/27/2018	1) Adding blood pressure monitoring instruction sheet from AHA<br>2) Add link to blood pressure monitoring video to Welcome letter<br>3) Updating text message content<br>4) Adding partner enrollment script/consent language<br>5) Protocol Changes: Updating Inclusion/Exclusion criteria	1) A .pdf infographic from the American Heart Association depicting proper home blood pressure monitoring<br>2) Adding link to a video on proper home blood pressure monitoring from Mayo Clinic<br>3) Edits to existing automated messaging around severely elevated BP measurements<br>4) Scripting to be used when contacting participant support partners<br>5) Updates to inclusion/exclusion criteria (2): increase from 2 visits in past year to two visits in two years; remove exclusion for liver cirrhosis	3/30/18

05/11/2018	1) Protocol change: updating exclusion criteria<br>2) Update Verbal consent script<br>3) Add/Remove study staff	1) Update to exclusion criteria: add exclusion for BMI >=50<br>2) Condensing >10 min script for better attention/comprehension by participant<br>3)Add Humphrey Chen, student research assistant. Remove Timothy McAuliffe, student research assistant.	05/22/2018
06/06/2018	1. Update baseline survey<br>2. Add new partner "welcome" message<br>3. Add new partner "thank you"/end of study message<br>4. Update provider notifications<br>5. Update number of recruitment calls<br>6. Update recruitment letter<br>7. Add study staff	1. To reduce confusion and increase consistence, the baseline medication adherence question was modified to match the positive framing of the weekly text message.<br>2. Added to confirm partner enrollment into Way to Health, confirm partner's ability to receive text messages, and to thank them for their participation.<br>3. Added to signal the end of their participation and to thank them for supporting their participant.<br>4. As a research project adjunct to clinical care, providers are notified when their patient is enrolled in the study. Additionally, since BP is being monitored remotely for hypertension management, PCPs are notified of out of control BP measurements so medications can be adjusted for better control.<br>5. Initial data extraction yielded a smaller than expected number of potentially eligible patients. We are increasing the number of recruitment call attempts from 3 to 5 to ensure sufficient sample size for the study.<br>6. As stated in the enrollment procedures, the participant will receive a mailed copy of the consent document upon enrollment. Removed erroneous language in the recruitment letter that referred to review of the "enclosed consent documents" since they are not being mailed until the participant has verbally consented and enrolled.<br>7. Add Jason Freeman, Research Coordinator.	6/19/18
08/08/18	Deviation: Non-participant mistakenly messaged when a consented participant's phone number was entered incorrectly into the text messaging platform.	New procedures were put into place for the recruiter to verbally confirm contact information while they were entering information from REDCAp into Way to Health, and to confirm receipt of the first "Welcome" text message by the study participant before ending the recruitment phone call to be sure that information was entered correctly.	8/13/18

08/10/18	1. Update of End of Study (EOS) Surveys (3: C, RM, RM+SS)<br>2. Updated Procedures: Clarification of monitoring activities<br>3. Use of additional BP monitor (Medline MDS4001) with XL sized cuff<br>4. Addition of End of Study Notification to providers<br>5. Addition of End of Study Reminder Letter to participants<br>6. Updated Background: Remove extraneous background information	1. Updated to match the language used in the baseline survey for medication adherence and forgetfulness. Adding three additional questions as appropriate: 1) to measure likeliness of recommending this program to a friend/family member to assess satisfaction and experience with the program, and 2) to measure the impact/influence of the support partner throughout the program.<br>2. Clarification of monitoring procedures<br>3. Accommodates participants with larger arms<br>4. Notify PCPs of participant's completion of the study<br>5. Providing text of letter reminding patients of EOS visit<br>6. Removed text inadvertently included when protocol document was created, is not related to Remote Monitoring for Hypertension. Text added was submitted with original IRB app but omitted in full study protocol.	08/22/18
08/31/18	Deviation: Two participants randomized to the social support study arm did not have the opportunity to receive feedback from their partners for about half the length of their participation in the study.	Error was identified during QI check. Once identified, the error was corrected immediately.	09/06/18
10/04/18	1. Increase sample size; update power calculation and effect size<br>2. Add additional text message reminder for end of study visits; update language to end of study visit reminder texts<br>3. Add new staff	1) Based on additional clinical information obtained after the study was initiated, we realized the study was not powered appropriately to determine the difference in SBP reduction planned. We are increasing our sample size from 150 to 250 as described above, to power the study sufficiently to be able to detect a larger difference between the control and intervention arms, and between the two intervention arms.<br>2) Increase EOS show rate	10/11/18
11/1/18	Continuing Review		11/6/18
11/30/18	1. Update provider notification language<br>2. Update provider notification procedures<br>3. Add patient reported blood pressure measurements<br>4. Update consent script	1) The language has been changed to inform providers that additional patient provided information is available for their review, and how to access that information if that patient is assigned to an intervention arm.<br>2) This change was made at the request of the providers at the Penn Family Care clinic to provide better clinical care to all participating patients.<br>3) Allows providers and others on the care team additional information that may be critical to decision making in the	12/20/18

		adjustment of medications and the overall care of the participating patients.<br>4) Addresses change in reporting to providers; only applicable to newly enrolled participants; existing participants not reconsented.	
06/12/19	Update Personnel	Add Kiernan McNelis – Research Assistant<br>Add Tamar Kleinman – Qualitative Research Specialist	6/14/19
07/19/19	Update Personnel	Add Caitlin McDonald – Clinical Research Coordinator	7/24/19
9/10/19	Clarify Analysis Plan	Before beginning any analysis, we felt it necessary to revisit outcomes and provide a more detailed/thorough analysis plan to ensure both were as complete as possible. The analysis plan was updated to be more descriptive of the specific comparisons that would be made, to include communication modality and demographics.	9/13/19
9/19/19	1) Update personnel<br>2) Update procedures<br>3) Clarify analysis plan	1) Add Chris Snider, Data Analyst<br>2) Chart review to document provider/care team actions after receiving monitoring encounters from research team to better understand the impact of the intervention on provider and care team time/workflow. Additionally added provider interviews.<br>3) Clarify passive follow-up period as 8 months post intervention	9/26/19
9/26/19	Update Personnel	1) Add Natalie Lee – Research Fellow	10/2/19
10/14/19	Update Personnel	1) Rebecca Anastos-Wallen – Research Analyst<br>2) Krisda Chaiyachati	10/17/19
11/12/219	Update Personnel	1) Danielle Mowry – Data Scientist<br>2) Corey Chivers – Data Scientist<br>3) Anahita Davoudi – Data Scientist	11/13/19
11/25/19	Update Personnel	1) Tim Delayney, Research Assistant	11/26/19
12/11/19	Update Personnel	1) Elizabeth Asch, Project Manager	12/17/19

Initial Statistical Analysis Plan

Analysis Plan

1.1 Power and sample size

Assuming 5.3mmHg standard deviation of SBP (given variability of BP over time) and a two-sided significance level of 0.017 (to accommodate the three pairwise comparisons), our sample size of 60 in each intervention arm and 30 in the control arm provides 80% power to detect a difference in SBP reduction of 3.75 mmHg between either intervention and usual care, and a difference in SBP reduction of 3.1 mmHg between the remote monitoring and social influence arms. A preliminary data extraction shows about 2300 patients uncontrolled hypertensive patients in PFC, which should provide a sufficient population for enrollment.

1.2 Data analysis

As a randomized controlled trial, we will conduct intention to treat analysis. For patients with missing data, we will first include blood pressure readings from the EMR and then conduct multiple imputations.

The primary outcome will relate the systolic blood pressure during the 4 month visit to intervention arm, adjusting for the initial systolic blood pressure by including baseline measures in the model. For patients with missing data who do not complete the 4 month in-person blood pressure reading, we will conduct multiple imputation of the SBP values at 4 months as a function of baseline variables, including intervention arm.

Secondary analyses include modeling of normotensive status using logistic regression; estimation of recruitment and attrition rates; and evaluation of patient perceptions about the interventions from follow-up surveys. We will also compare medication adherence by intervention arm after enrollment. We will adjust these analyses for BMI, age, gender, race, forgetfulness (from the baseline and follow-up survey), and diabetic status. In addition, we will track BP measurements from the EMR that are obtained through usual care as well as during PCP visits, MPM messaging, phone calls; hospitalizations; and medication changes for up to 8 months after the study intervention using similar models. Lastly we will analyze diastolic blood pressure at the 4-month visit, adjusting for the initial diastolic blood pressure.

Final Statistical Analysis Plan

****New changes from initial protocol notated in bold, parts removed from initial protocol notated in strikethrough**

Analysis Plan

<u>1.1 Power and sample size</u>

Assuming 5.3mmHg standard deviation of SBP (given variability of BP over time) and a two-sided significance level of 0.017 (to accommodate the three pairwise comparisons), our sample size of 60 in each intervention arm and 30 in the control arm provides 80% power to detect a difference in SBP reduction of 3.75 mmHg between either intervention and usual care, and a difference in SBP reduction of 3.1 mmHg between the remote monitoring and social influence arms. A preliminary data extraction shows about 2300 patients uncontrolled hypertensive patients in PFC, which should provide a sufficient population for enrollment. **However, based on additional clinical information obtained after the study was initiated, we estimate a standard deviation for change in SBP of 20mmHg, larger than our initial estimate. Thus, we increased our accrual target to 100 in each intervention arm and 50 in the control arm, which provides 80% power to detect a difference in SBP reduction of 11mmHg between either intervention and usual care, and a difference in SBP reduction of 9mmHg between the remote monitoring and social influence arms.**

<u>1.2 Data analysis</u>

As **is standard in** a randomized controlled trial, ~~we will conduct~~**our primary analysis will follow the** intention to treat **approach, in which participants are analyzed according to their assigned treatment regardless of compliance. analysis.** For patients with missing **blood pressure** data, we will first include ~~blood pressure~~ readings **available** from the **electronic health record (**E**H**~~M~~R**) occurring from 90-150 days from the participant's start in the intervention,** and then conduct multiple imputation **using all available baseline covariates**.

The primary ~~outcome~~ **analysis** will relate the systolic blood pressure ~~during~~**at** the 4 month visit to **assigned** intervention arm, adjusting for the initial systolic blood pressure by including **the** baseline measures in the model. ~~For patients with missing data who do not complete the 4 month in-person blood pressure reading, we will conduct multiple imputation of the SBP values at 4 months as a function of baseline variables, including intervention arm.~~

Secondary analyses include ~~modeling~~ **analyzing achievement** of normotens**ion**~~ive status~~**by study arm** using logistic regression **and repeating the analyses for systolic blood pressure with diastolic blood pressure at the 4-month visit. We will** estimat**e**~~ion of~~ recruitment and attrition rates, and evaluat**e**~~ion of~~ patient perceptions about the interventions from follow-up surveys. We will also compare medication adherence **and blood pressure monitoring adherence** by intervention arm after enrollment. We will adjust these analyses for BMI, age, gender, race, forgetfulness (from the baseline and follow-up survey), and diabetic status. In addition, we will track BP measurements **by arm** from the **(**E**H**~~M~~R**)** that are obtained through usual care **for up to 8 months after the study intervention. We will** ~~as well as during~~**also evaluate** PCP visits, MPM messaging, phone calls, hospitalizations, and medication changes. ~~for up to 8 months after the~~

~~study intervention using similar models. Lastly we will analyze diastolic blood pressure at the 4-month visit, adjusting for the initial diastolic blood pressure.~~**We will repeat all analyses by sub-group related to time of enrollment (round 1 and round 2). Finally, we will perform a thematic evaluation of provider perceptions about the interventions from information gathered during follow-up telephone interviews as well as process data collected from a review of provider actions following monitoring encounters.**

Summary of Statistical Analysis Plan Modifications

Before beginning any analysis, we felt it necessary to revisit outcomes and provide a more detailed/thorough analysis plan to ensure both were as complete as possible. The analysis plan was updated to be more descriptive of the specific comparisons that would be made, to include communication modality and demographics.

Appendix A: Final Interview Guides

Support BP Interview Protocol – Provider

**Indented bullet points are potential probes

<u>Introduction/Description</u>

SupportBP is a study comparing the effectiveness of different approaches to improving blood pressure control amongst patients with hypertension. The study used remote monitoring of blood pressure readings and medication adherence through text messaging as well as the inclusion of a support partner engaged in "facilitated cheerleading" to provide motivation to the patient. The questions I would like to ask focus on the provider's experience with SupportBP. I will ask you to talk about your personal response to SupportBP encounters and your overall perception of the intervention from a provider perspective. Our intention is to improve the program for future iterations based on both patient and provider feedback.

Your participation in this study is confidential. With your permission, this interview will be recorded to accurately keep track of information, but the recording will be deleted at the completion of the study. Your participation in this study is important. However, if you would wish to stop at any time, you may do so without any consequence to you. Additionally, feel free to ask me any questions concerning the interview or the study as we proceed. May we begin?

<u>Clinical Care Background for Hypertension Patients</u>

- Describe how you typically manage the blood pressure of your hypertensive patients.
    - Additional probes: How often do you have hypertension patients return for visits? Do you ask patients to monitor their blood pressure at home? Do you give patients a blood pressure cuff during the appointment or are they responsible for acquiring one? Do patients log their blood pressure measurements over time?
- What is the patient's role in managing hypertension?
    - Rephrase: What are your expectations of your patients in regard to monitoring their blood pressure?
- How do patients track their blood pressure outside of office visits and how do the patients communicate this information to you?
    - Rephrase: How do you communicate with your patients regarding their blood pressure monitoring and medication adherence?
- What criteria do you use to decide when to elevate a patient's blood pressure medication?

<u>Overall Perceptions of SupportBP</u>

- What are your overall perceptions of SupportBP?
- Tell me about the impact of SupportBP on your patient's blood pressure control.
- How did this program affect your work flow and clinical practice?
- How do you want to receive this information? Do you want to receive the encounters or would you prefer that someone else receives it?
- How much do you want the program to manage and do in terms of adjusting medication and communicating with the patient?
    - How much involvement would you as the physician prefer to have?

Future Improvements

- How can this program be improved for the patients?
- How can this program be improved from providers?
  - If we don't want to separate question for patients/providers: In future iterations of this intervention, what improvements would you make?
- Please name three things you like and dislike about SupportBP.

Support BP Interview Protocol – Nurse Pool

**Indented bullet points are potential probes

Introduction/Description

SupportBP is a study comparing the effectiveness of different approaches to improving blood pressure control amongst patients with hypertension. The study used remote monitoring of blood pressure readings and medication adherence through text messaging as well as the inclusion of a support partner engaged in "facilitated cheerleading" to provide motivation to the patient. The questions I would like to ask focus on the provider's experience with SupportBP. I will ask you to talk about your personal response to SupportBP encounters and your overall perception of the intervention from a provider perspective. Our intention is to improve the program for future iterations based on both patient and provider feedback.

Your participation in this study is confidential. With your permission, this interview will be recorded to accurately keep track of information, but the recording will be deleted at the completion of the study. Your participation in this study is important. However, if you would wish to stop at any time, you may do so without any consequence to you. Additionally, feel free to ask me any questions concerning the interview or the study as we proceed. May we begin?

SupportBP Encounter Response

- Describe the process of response to a SupportBP encounter.
    - Additional probes: Who typically responded to the encounter? What steps were taken when an encounter was received? Was the patient contacted after an encounter was received, and, if so, how was this communication structured?
- How was communication between the nurse pool structured?
- Was there communication with the patient's physician throughout the intervention?
    - At what point was the physician looped into the response, if at all?
- How much time did these responses require each daily/weekly?

Overall Perceptions of SupportBP

- What are your overall perceptions of SupportBP?
- Tell me about the impact of SupportBP on your patient's blood pressure control.
- How did this program affect your work flow?
- How do you want to receive this information? Do you want to receive the encounters or would you prefer that someone else receives it?
- How do you feel about the number of people that were responsible for responding to SupportBP encounters?
    - Rephrase: Would you say that the number of people in the nurse pool was sufficient for effectively responding to the encounters?

Future Improvements

- How can this program be improved for the patients?
- How can this program be improved from providers?
    - If we don't want to separate question for patients/providers: In future iterations of this intervention, what improvements would you make?
- Please name three things you like and dislike about SupportBP.

Support BP Interview Protocol – Nurse Practitioner

**Indented bullet points are potential probes

Introduction/Description

SupportBP is a study comparing the effectiveness of different approaches to improving blood pressure control amongst patients with hypertension. The study used remote monitoring of blood pressure readings and medication adherence through text messaging as well as the inclusion of a support partner engaged in "facilitated cheerleading" to provide motivation to the patient. The questions I would like to ask focus on the provider's experience with SupportBP. I will ask you to talk about your personal response to SupportBP encounters and your overall perception of the intervention from a provider perspective. Our intention is to improve the program for future iterations based on both patient and provider feedback.

Your participation in this study is confidential. With your permission, this interview will be recorded to accurately keep track of information, but the recording will be deleted at the completion of the study. Your participation in this study is important. However, if you would wish to stop at any time, you may do so without any consequence to you. Additionally, feel free to ask me any questions concerning the interview or the study as we proceed. May we begin?

Clinical Care Background for Hypertension Patients

- Describe how you typically manage the blood pressure of your hypertensive patients.
  - Additional probes: How often do you have hypertension patients return for visits? Do you ask patients to monitor their blood pressure at home? Do you give patients a blood pressure cuff during the appointment or are they responsible for acquiring one? Do patients log their blood pressure measurements over time?
- What is the patient's role in managing hypertension?
  - Rephrase: What are your expectations of your patients in regard to monitoring their blood pressure?
- How do patients track their blood pressure outside of office visits and how do the patients communicate this information to you?
  - Rephrase: How do you communicate with your patients regarding their blood pressure monitoring and medication adherence?
- What criteria do you use to decide when to elevate a patient's blood pressure medication?

Overall Perceptions of SupportBP

We will ask about your perceptions of both the first round of the program as a provider with patients enrolled in the study and the second round with the encounters routed to the nursing pool.

First Round – Provider

- What are your overall perceptions of SupportBP in the first round prior to the encounters being routed to the nursing pool?
- How did this program affect your work flow and clinical practice in the first round?
- Tell me about the impact of SupportBP on your patient's blood pressure control in the first round.
- How do you want to receive this information as a provider?
- How much do you want the program to manage and do in terms of adjusting medication and communicating with the patient?
  - How much involvement would you as the provider prefer to have

Second Round – Nursing Pool

- What are your overall perceptions of SupportBP in the second round with the encounters being routed to the nursing pool?
- How did this program affect your work flow and clinical practice in the second round?
- Tell me about the impact of SupportBP on your patient's blood pressure control.
- Do you want to continue to receive the encounters or would you prefer that someone else receives it?
- Comparing first round to second round, what are your perceptions of how other physicians regarded the program with the change in workflow to direct all encounters to the nursing pool?
- What could be improved regarding communication between the clinicians and the SupportBP program?

Future Improvements

- How can this program be improved for the patients?
- How can this program be improved for providers?
- What would need to be changed about SupportBP in order to be a clinically practical intervention using the nursing pool model?
- Please name three things you like and dislike about SupportBP.

Additional Questions for Dr. Teel – Vice Chair for Clinical Operations

Frame: unique position so we want to ask you some specific questions addressing that position. Asking based on all of the feedback that he heard.

- What was the general perception of SupportBP amongst the physicians in the practice?
  - Would you say that most clinicians had a positive or negative experience with SupportBP?
- How did the perception of SupportBP change after switching to the nurse pool model?
- What could be improved regarding communication between the clinicians and the SupportBP program?
- What would need to be changed about SupportBP in order to be a clinically practical intervention based on clinician feedback?

Appendix B: Final Text Message Content

Weekly Text Messaging Content (Repeated Weekly)

	Day 1	Day 2	Day 3	Day 4	Day 5	Day 6	Day 7
**BP Measurement**[1]	X		X		X		
**BP Feedback**						X	
**Medication Adherence**				X			
**Medication Feedback**				X			
**Support Partner Msg** (to partner, SS arm only)							X
**Support Partner Feedback** (to Patient, SS arm only)							X

[1] *BP Measurement prompts sent at 8am. If no response by 7pm, a reminder was sent. Patients had 24 hours to respond to each prompt.*

Welcome
Arms: as below
Begin: Day 0
Repeats on days: does not repeat
At: upon randomization

- Arms 1 & 2:
    - "Welcome to SupportBP, PARTICIPANT_FIRSTNAME! You'll receive your welcome packet, blood pressure cuff & instructions within about a week, and your first texts will start shortly after that. Questions? Call 215-662-8956 or email SupportBP@uphs.upenn.edu"
- Arm 3:
    - "Welcome to SupportBP, PARTICIPANT_FIRSTNAME! You'll receive your welcome letter within about a week. Questions? Call 215-662-8956 or email SupportBP@uphs.upenn.edu"

BP Reminders
Arms: 1& 2
Begin: Day 8
Repeats on days: 8, 10, 12, 15, 17, 19, 22, 24, 26, 29, 31, 33, 36, 38, 40, 43, 45, 47, 50, 52, 54, 57, 59, 61, 64, 66, 68, 71, 73, 75, 78, 80, 82, 85, 87, 89, 92, 94, 96, 99, 101, 103, 106, 108, 110, 113, 115, 117
At: 8am

- "What is your blood pressure today? (Ex. 120/80)
    - If valid input <180/110 received, "{systolic/diastolic} has been recorded. Thank you."
    - If valid input >180/110 received, "Your blood pressure ({systolic_value}/{diastolic_value}) is elevated. Please wait 15 minutes and check it again. If the top number is still higher than 180 -OR- the bottom number is still higher than 110, please come in to the office (Penn Family Care: 3737 Market St., 9th Floor, Philadelphia, PA 19104) for a blood pressure check with a nurse during normal office hours. " *

    - If input format incorrect, “Sorry, we do not understand that response. Please input BP in the following format: 120/80. Questions? Call 215-662-8956.”
    - If no input received, no response.
- * An alert will also be sent to the Research Coordinator to follow-up with participant by phone and alert the patient’s PCP via PennChart.

BP Feedback
Arms: 1&2
Begin: Day 13
Repeats on days: Day 13 + every 7 days: 13, 20, 27, 34, 41, 48, 55, 62, 69, 76, 83, 90, 97, 104, 111, 118
At: 10:30am

- Looks at last 3 BP Reminder responses:
    - (if 3 of 3) “You submitted 3 out of 3 BP measurements this week, great job and keep it up!”
    - (if 1-2 of 3) “You submitted X out of 3 BP measurements this week, thank you for monitoring. Next week, aim for 3 times!”
    - (if 0 of 3), “We have not received any BP measurements from you this week, it’s important that you monitor if your pressure is controlled. Questions? Call 215-662-8956.

Medication Adherence
Arms: 1&2
Begin: Day 4
Repeats on days: Day 4 + every 7 days: 4, 11, 18, 25, 32, 39, 46, 53, 60, 67, 74, 81, 88, 95, 102, 109, 116
At: 8am

- “How many days did you take your blood pressure medication(s) in the past 7 days? (Please input a single number: 0-7)”

Medication Feedback
Arms 1&2:
Begin: Day 4
Repeats on days: Day 4 + every 7 days: 4, 11, 18, 25, 32, 39, 46, 53, 60, 67, 74, 81, 88, 95, 102, 109, 116
At: upon response to Medication Adherence text, or after 8 hrs with no response

- Looks at last Medication Adherence response:
    - (if 7 of 7, respond) “Great! You reported taking your BP meds 7 of the last 7 days. Keep up the good work of taking your medication every day!”
    - (if 4-6 of 7, respond) “Thank you. You reported taking your BP meds X of the last 7 days. Next week, aim for all 7 days for better blood pressure control.”
    - (if 0-3 of 7) “You reported taking your BP meds X of the last 7 days. Taking your medication every day is an important step in controlling your blood pressure. Next week, aim to take them daily.”
    - If input format incorrect, “Sorry, we do not understand that response. Please input a number: 0-7. Questions? Call 215-662-8956.”

- If no input received, ”You did not report taking your medication at all this week. Taking your medication every day is an important step in controlling your blood pressure. Next week, aim to take them daily.”

Partner Welcome Message
Arms: 2 (to partner only)
Begin: Day 0
Repeats: does not repeat
At: upon enrollment

- “Welcome to SupportBP! Thank you for participating in support of <participant_name>. You’ll receive your first text message in about 8-10 days. Questions? Please call 215-662-8956 or email SupportBP@uphs.upenn.edu.”

Partner Message
Arms: 2 (to partner only)
Begin: Day 14
Repeats on Days: Day 14 + every 7 days: 14, 21, 28, 35, 42, 49, 56, 63, 70, 77, 84, 91, 98, 105, 112, 119
At: 8am

- If participant (BP=3) and (Med=7)
    - “<Participant Name> checked BP 3 of 3 times and meds 7 of 7 times this past week. We’ll let them know you think they’re doing great, unless you text “No” in reply to this message within the next 2 hours.”
- If participant (1>BP>3) and ((1>Med>7))
    - “<Participant Name> checked BP X out of 3 times and took their meds X out of 7 times this week. We’ll let them know you think they could do better, unless you text “No” in reply to this message within the next 2 hours.”
- If participant (BP=3) and ((Med<7))
    - “<Participant Name> checked BP 3 of 3 times and meds X of 7 times this week. We’ll let them know you want them to take them all 7 days next week, unless you text “No” in reply to this message within the next 2 hours.”

- If participant (BP<3) and (Med=7)
    - “<Participant Name> checked BP X of 3 times and meds 7 of 7 times this week. We’ll let them know you want them to better monitor their BP next week unless you text “No” in reply to this message within the next 2 hours.”

- If participant (BP=0) and ((Med=0))
    - “<Participant Name> did not report checking their BP and took their 0 of 7 times this week, we’ll let them know you think it’s important they start taking better care, unless you text “No” in reply to this message within the next 2 hours.”
- If participant (BP=0) and (Meds>0)
    - “<Participant Name> did not report checking their BP at all this week, and reported taking their BP meds X of 7 times. We’ll let them know you think it’s important they start taking better care, unless you text “No” in reply to this message within the next 2 hours.

- If participant (BP>0) and (Meds = missing)
    - "<Participant Name> checked their BP X out of 3 times, and did not report taking their BP meds at all this past week. We'll let them know you think it's important they start taking better care, unless you text "No" in reply to this message within the next 2 hours.

- If support partner responds to any of the above with "No," then respond, "Thank you, we will not send a message to <participant name> today on your behalf. Please be sure to support <participant name> in regular BP monitoring and daily medication adherence."

- If support partner responds to any of the above with invalid input, then respond, "Sorry, we do not understand that response. Please only respond with "No" if you do not want us to send a message to <participant>. Otherwise, no response is necessary. Questions? Call 215-662-8956."

Partner Feedback
Arms: 2 only
Begin: Day 14
Repeats on: Day 14 + every 7 days: 14, 21, 28, 35, 42, 49, 56, 63, 70, 77, 84, 91, 98, 105, 112, 119
At: after 2hrs of non-response from partner

- If participant (BP=3) and (Med=7) and no response from partner after 2 hours:
    - "Your support partner says, "Great job monitoring your BP and taking your meds! Keep it up!'"
- If participant (BP=1-2) and (Med=0-6) and no response from partner after 2 hours:
    - "Your support partner says, 'Good job monitoring your BP and taking your meds, but you could do better with both. Next week aim for 3 readings and 7 days of meds!'"

- If participant (BP=3) and (Med <7) and no response from partner after 2 hours:
    - "Your support partner says, "Great job monitoring your BP this week! Keep it up! You could still do better with your meds. Next week, aim for all 7 days of your medication.'"

- If participant (BP<3) and (Med=7) and no response from partner after 2 hours:
- "Your support partner says, "Great job taking your meds this week! Keep it up! You could still do a better job monitoring your BP. Next week, aim for 3 BP readings.'"
    -
- If participant (BP=0) and ((Med=0)) and no response from partner after 2 hours:
- "Your support partner says, 'Please remember to monitor your BP and take your meds next week. It's important to me that you get your blood pressure under control because it's important to your health!'"
  If participant (BP=0) and (Meds=missing) and no response from partner after 2 hours:
    - "Your support partner says, 'Please remember to monitor your BP and take your meds next week. It's important to me that you get your blood pressure under control because it's important to your health."

- If participant (BP=0) and (Meds>0) and no response from partner after 2 hours:

    - “Your support partner says, Please remember to monitor your BP and keep up taking your meds next week. It’s important to me that you get your blood pressure under control because it’s important to your health.”

- If participant (BP>0) and (Meds=missing) and no response from partner after 2 hours:
    - “Your support partner says, ‘Please keep up monitoring your BP and remember to take your meds next week. It’s important to me that you get your blood pressure under control because it’s important to your health.”

Week 1 (only) Partner Message
Arms: 2 (to partner only)
Begin: Day 7
Repeats on Days: does not repeat
At: (time)

- If participant (Med=7)
    - “<Participant Name> took their BP meds 7 of 7 times this past week. We’ll let them know you think they’re doing great, unless you text “No” in reply to this message within the next 2 hours.”

- If participant (1>Med<7)
    - “<Participant Name> took their BP meds X out of 7 times this week. We’ll let them know you think they could do better, unless you text “No” in reply to this message within the next 2 hours.”

- If participant (Med=0)
    - “<Participant Name> did not take their meds this week, we’ll let them know you think it’s important they start taking better care, unless you text “No” in reply to this message within the next 2 hours.”
- If participant did not respond to medication adherence text, “<Participant name> did not reporting taking any medication(s) this week. We’ll let them know you think it’s important they start taking them regularly, unless you text “No” in reply to this message within the next 2 hours.

- If support partner responds to any of the above with “No,” then respond, “Thank you, we will not send a message to <participant name> today on your behalf. Please be sure to support <participant name> in regular BP monitoring and daily medication adherence.”

- If support partner responds to any of the above with invalid input, then respond, “Sorry, we do not understand that response. Please only respond with “No” if you do not want us to send a message to <participant>. Otherwise, no response is necessary.”

Week 1 (only) Partner Feedback
Arms: 2 only
Begin: Day 7
Repeats on: does not repeat
At: (time)

- If participant (Med=7) and no response from partner after 2 hours:
  - Your support partner says, "Great job taking your meds! Keep it up!'"
- If participant (1>Med>7) and no response from partner after 2 hours:
  - "Your support partner says, 'Good job taking your meds this week, but I think you could do better. Next week aim for all 7 days of meds!'"

If participant (Med=0) and no response from partner after 2 hours:

  - "Your support partner says,says, 'Please take your meds next week. It's important to me that you get your blood pressure under control because it's important to your health!'"
- If participant did not respond to medication adherence text, "Your support partner says,says, 'You didn't report taking any BP meds this week. Please work on taking them daily. It's important to me that you get your blood pressure under control because it's important to your health."

End of Study Visit
Arms: 1, 2, 3
Begin: Day (End of Study date – 2 weeks)
Repeats on: (EOS date – 3 days and EOS date – 1 day)
At: 8am

- "Reminder: <Participant_First_Name>, your in-office blood pressure check is scheduled for <time> on <date> at at Penn Family Care, 3737 Market St. Philadelphia, PA. 9th Floor, Reception. Completing this 15min visit earns you $50! Need to reschedule? Call: 215-662-8956.

Note these begin/repeat dates will change as EOS visit gets rescheduled, participants may receive more or less than 3 reminders.

Arm: 2 (partner only)
Begin: after participant completion of the EOS visit/participant study close-out
Repeats: does not repeat
At: participant study close out

- "Thank you for supporting <participant_name> through the SupportBP study. You will no longer receive these text messages, but we encourage you to continue to support <participant_name> in managing their blood pressure."

End of Program
Arms: 1&2
Begin: Day 119
Repeats on: does not repeat
At: 8am

- "Thank you for your participation in Support BP! You will no longer receive blood pressure reminders or feedback."
