# Supplementary material for: Remote Blood Pressure Monitoring With Social Support for Patients With Hypertension: A Randomized Clinical Trial
Source: JAMA Netw Open. 2024 Jun 3;7(6):e2413515. doi: 10.1001/jamanetworkopen.2024.13515 (PMC11148689; doi:10.1001/jamanetworkopen.2024.13515)

Supplemental Online Content

Mehta SJ, Volpp KG, Troxel AB, et al. Remote blood pressure monitoring with social support for patients with hypertension: a randomized controlled trial. *JAMA Netw Open*. 2024;7(6):e2413515. doi:10.1001/jamanetworkopen.2024.13515

eTable 1. End of Study BP (Complete Cases Only, n=206)

eTable 2. Adherence to BP Monitoring (Complete Cases Only)

eTable 3. Mean Visit Utilization During and After BP Monitoring

eTable 4. Change in Systolic BP Between Baseline and End of Study Adjusted for Baseline Systolic BP Among Phase 1 (2018) and Phase 2 (2019) Participants

eTable 5. Days Elapsed from Enrollment Through End of Study Visit

eTable 6. Self-Reported Frequency of BP Monitoring and Medication Adherence at Baseline and End of Study (Paired Cases Only, n=209)

eTable 7. Participant Experience (Completed Patients in RM or SS Group)

eFigure. Changes in BP Distribution from Baseline to End of Study (Complete Cases Only)

This supplemental material has been provided by the authors to give readers additional information about their work.

eTable 1. End of Study BP (Complete Cases Only, n=206) [1]

Coefficient	Estimate (95% CI)	p-value
**Systolic BP (mmHg)**		
(Intercept)	83.36 (53.85 – 112.87)	
Arm RM	-4.12 (-9.74 -1.51)	0.15
Arm SS	-0.19 (-5.78 - 5.40)	0.95
Arm C	Reference	
Baseline Systolic BP	0.35 (0.16 - 0.54)	<0.001
**Diastolic BP (mmHg)**		
(Intercept)	42.98 (31.82 - 54.15)	
Arm RM	-1.16 (-4.31 - 1.99)	0.47
Arm SS	-0.24 (-3.38 - 2.90)	0.88
Arm C	Reference	
Baseline Diastolic BP	0.47 (0.35 - 0.59)	<0.001

[1] *Adjusted for baseline systolic/diastolic BP*

eTable 2. Adherence to BP Monitoring (Complete Cases Only)

	Study Arm		p-value
	**RM**	**SS**	
**N**	84	87	
Percent of expected measurements received, mean (SD)	76.5 (19.8)	77.2 (21.8)	.83

eTable 3. Mean Visit Utilization During and After BP Monitoring

		Monitoring Period (0-4 Months)			Post-Monitoring Period (5-12 months)		
	**N**	**PCP Visits**	**ED Visits**	**Hospital Admissions**	**PCP Visits**	**ED Visits**	**Hospital Admissions**
**RM**	100	1.14	0.11	0.04	2.06	0.13	0.06
**SS**	97	1.37	0.15	0.22	2.38	0.22	0.11
**Control**	49	1.04	0.12	0.0	1.69	0.10	0.06
**Overall**	246	1.21	0.13	0.04	2.11	0.16	0.08

eTable 4. Change in Systolic BP Between Baseline and End of Study Adjusted for Baseline Systolic BP Among Phase 1 (2018) and Phase 2 (2019) Participants

**Phase 1 (2018)**		
**Coefficient**	**Estimate (95% CI)**	**p-value**
Systolic BP (mmHg)		
(Intercept)	89.08 (50.95 - 127.20)	
RM	-4.49 (-12.44 – 3.45)	0.25
RM + SS	-0.81 (-8.99 – 7.37)	0.84
Control	Reference	
Baseline Systolic BP	0.32 (0.07 – 0.57)	0.01
*Adjusted for baseline systolic BP		
**Phase 2 (2019)**		
**Coefficient**	**Estimate (95% CI)**	**p-value**
Systolic BP (mmHg)		
(Intercept)	87.54 (44.10 – 130.97)	
RM	-4.56 (-13.13 – 4.01)	0.29
RM + SS	-0.28 (-9.42 – 8.87)	0.95
Control	Reference	
Baseline Systolic BP	0.31	0.04
*Adjusted for baseline systolic BP		

eTable 5. Days Elapsed from Enrollment Through End of Study Visit[1]

**Phase 1**	**RM (n=49)**	**RM+SS (n=53)**	**Control (n=22)**	**Total (n=124)**
# Days, Mean (SD)	124.8 (7.8)	123.4 (6.8)	130.7 (6.4)	125.3 (7.5)
# Days, Median (IQR)	124 (121.127)	122 (120-124)	129 (126-136)	124 (120.5-128)
# Days, Min-Max	107-149	110-150	120-144	107-150
**Phase 2**	**RM (n=35)**	**RM+SS (n=36)**	**Control (n=15)**	**Total (n=86)**
# Days, Mean (SD)	125.7 (6.9)	120.9 (6.9)	128.7 (9.2)	124.2 (7.9)
# Days, Median (IQR)	124 (120-129)	119.5 (116-122.5)	125 (122-132)	122 (120-127)
# Days Min/Max	117-147	110-139	120-148	110-148
**Combined**	**RM (n=84)**	**RM+SS (n=89)**	**Control (n=37)**	**Total (n=210)**
# Days, Mean (SD)	125.2 (7.4)	121 (6.9)	129.9 (7.6)	124.8 (7.7)
# Days, Median (IQR)	124 (120-127)	121 (119-124)	127 (125-134)	123 (120-128)
# Days Min/Max	107-149	110-150	120-148	107-150

eTable 6. Self-Reported Frequency of BP Monitoring and Medication Adherence at Baseline and End of Study (Paired Cases Only, n=209)

Study Arm		BP Frequency, Median (IQR)		Med Adherence, Median (IQR)		Difficulty Remembering to Take BP Meds[2] N (%)	
	N	Baseline	EOS	Baseline	EOS	Baseline	EOS
**RM**	84	0 (0-2)	9 (4-14)	14 (12-14)	14 (13-14)	35 (41.7)	23 (27.4)
**SS**	88	0 (0-2)	8 (6-14)	14 (13-14)[1]	14 (14-14)	28 (31.8)	17 (19.3)
**C**	37	0 (0-0)	0 (0-2)	14 (12-14)	14 (13-14)	15 (40.5)	16 (43.2)

[1] *Missing one survey value*

[2] *Participants reporting "Sometimes, Often, or Always"*

eTable 7. Participant Experience (Completed Patients in RM or SS Group)

	**RM**	**SS**
**Strongly Agree/Agree program helpful in remembering to monitor BP,** N (%)	81/84 (96.4%)	78/88 (88.6%)
**Strongly Agree/Agree program helpful in remembering to take BP medications,** N (%)	67/84 (80%)	66/88 (75%)
**Strongly Agree/Agree partner feedback was helpful in remembering to monitor BP,** N (%)	n/a	67/88 (76.1%)
**Very likely to recommend program to a friend or family member who needed it,** N (%)[1]	77/84 (91.7%)	61/85 (72%)
**Net Promoter Score**	90.5	69.4

[1] *Participants responding 9 or 10 on a scale from 0-10 with 0 being very unlikely to recommend*

eFigure. Changes in BP Distribution from Baseline to End of Study (Complete Cases Only)

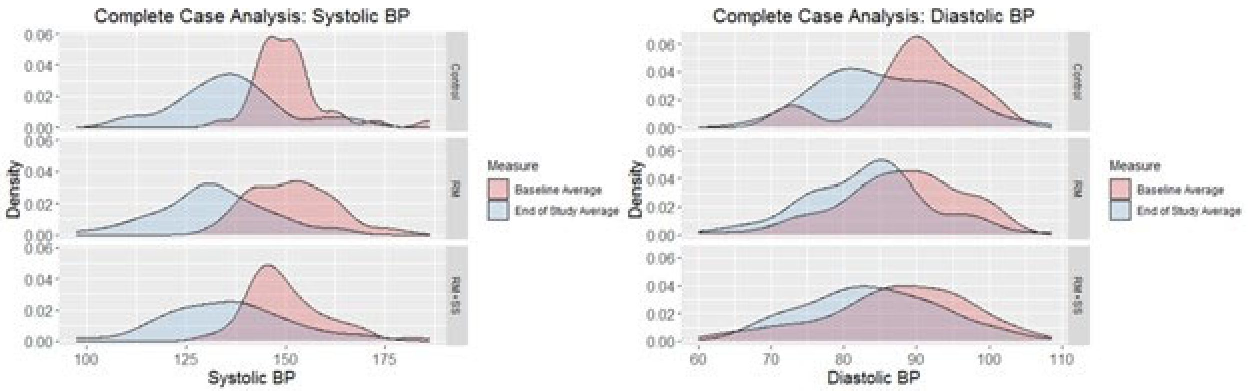

Supplement: Supplement 2. — eTable 1. End of Study BP (Complete Cases Only, n = 206) eTable 2. Adherence to BP Monitoring (Complete Cases Only) eTable 3. Mean Visit Utilization During and After BP Monitoring eTable 4. Change in Systolic BP Between Baseline and End of Study Adjusted for Baseline Systolic BP Among Phase 1 (2018) and Phase 2 (2019) Participants eTable 5. Days Elapsed from Enrollment Through End of Study Visit eTable 6. Self-Reported Frequency of BP Monitoring and Medication Adherence at Baseline and End of Study (Paired Cases Only, n = 209) eTable 7. Participant Experience (Completed Patients in RM or SS Group) eFigure. Changes in BP Distribution from Baseline to End of Study (Complete Cases Only) [file jamanetwopen-e2413515-s002.pdf]
