# Supplementary material for: Remote Blood Pressure Monitoring With Social Support for Patients With Hypertension: A Randomized Clinical Trial
Source: JAMA Netw Open. 2024 Jun 3;7(6):e2413515. doi: 10.1001/jamanetworkopen.2024.13515 (PMC11148689; doi:10.1001/jamanetworkopen.2024.13515)
Supplement: Supplement 3. — Data Sharing Statement [file jamanetwopen-e2413515-s003.pdf]

Data Sharing Statement

Mehta. Remote Blood Pressure Monitoring With Social Support for Patients with Hypertension. *JAMA Netw Open*. Published June 03, 2024. doi:10.1001/jamanetworkopen.2024.13515

Data
Data available: No
